# A glutamine tug-of-war between cancer and immune cells: recent advances in unraveling the ongoing battle

**DOI:** 10.1186/s13046-024-02994-0

**Published:** 2024-03-08

**Authors:** Bolin Wang, Jinli Pei, Shengnan Xu, Jie Liu, Jinming Yu

**Affiliations:** 1 Lung Cancer Center, West China Hospital, Sichuan University, Chengdu, 610041 China; 2 Department of Radiation Oncology and Shandong Provincial Key Laboratory of Radiation Oncology, Shandong Cancer Hospital and Institute, Shandong First Medical University and Shandong Academy of Medical Sciences, Jinan, Shandong China; 3 Research Unit of Radiation Oncology, Chinese Academy of Medical Sciences, Jinan, Shandong China

**Keywords:** Glutamine metabolism, Cancer, Immune cells, Tumor microenvironment, Therapeutic strategies

## Abstract

Glutamine metabolism plays a pivotal role in cancer progression, immune cell function, and the modulation of the tumor microenvironment. Dysregulated glutamine metabolism has been implicated in cancer development and immune responses, supported by mounting evidence. Cancer cells heavily rely on glutamine as a critical nutrient for survival and proliferation, while immune cells require glutamine for activation and proliferation during immune reactions. This metabolic competition creates a dynamic tug-of-war between cancer and immune cells. Targeting glutamine transporters and downstream enzymes involved in glutamine metabolism holds significant promise in enhancing anti-tumor immunity. A comprehensive understanding of the intricate molecular mechanisms underlying this interplay is crucial for developing innovative therapeutic approaches that improve anti-tumor immunity and patient outcomes. In this review, we provide a comprehensive overview of recent advances in unraveling the tug-of-war of glutamine metabolism between cancer and immune cells and explore potential applications of basic science discoveries in the clinical setting. Further investigations into the regulation of glutamine metabolism in cancer and immune cells are expected to yield valuable insights, paving the way for future therapeutic interventions.

## Introduction

Over the past two decades, remarkable progress has been made in the field of cancer metabolism, attracting widespread attention [1]. This area of research has been inspired by Otto Warburg's observations in 1922, wherein he discovered the specific glucose consumption by cancer cells through the Warburg metabolic pathway [2]. Specifically, glucose-lactate metabolism in tumor tissues increased significantly by tenfold compared to normal tissues under aerobic conditions, highlighting the differences between cancer cells and normal cells [2]. This pivotal discovery not only serves as the foundation for fluro-18-deoxyglucose positron emission tomography-computed tomography imaging but also emphasizes the significance of investigating cancer metabolism [3]. Further investigations have revealed that immune cells tend to preferentially uptake glucose, while cancer cells exhibit a greater affinity for glutamine absorption in various cancer models [4]. This finding has prompted deeper contemplation regarding the significance of glutamine metabolism in cancer and immune cell research [5]. Particularly within the tumor microenvironment (TME), the competition for nutrients and utilization of glutamine between cancer cells and immune cells has become a focal point of study [6]. The accumulating evidence strongly emphasizes the critical role of glutamine metabolism in the intricate interplay between cancer cells and immune cells [7]. Delving deeper into the importance of glutamine metabolism will further propel our understanding of the relationship between cancer and immune cells.

In this review, we provide a comprehensive overview of the recent advances in unraveling the tug-of-war of glutamine metabolism between cancer and immune cells. We discuss update of recent efforts to the molecular mechanisms involved in glutamine uptake, metabolism, and its impact on cancer cell proliferation and immune cell function. Furthermore, we review the therapeutic strategies aimed at targeting glutamine metabolism as a means to enhance anti-tumor immune responses, as well as current knowledge gaps and opportunities. The insights gained from this review will contribute to the development of novel therapeutic approaches aimed at tipping the balance in favor of the immune system and improving patient outcomes.

### Glutamine emerges as a rising star in the field of cancer immunotherapy

**Fig. 1:**
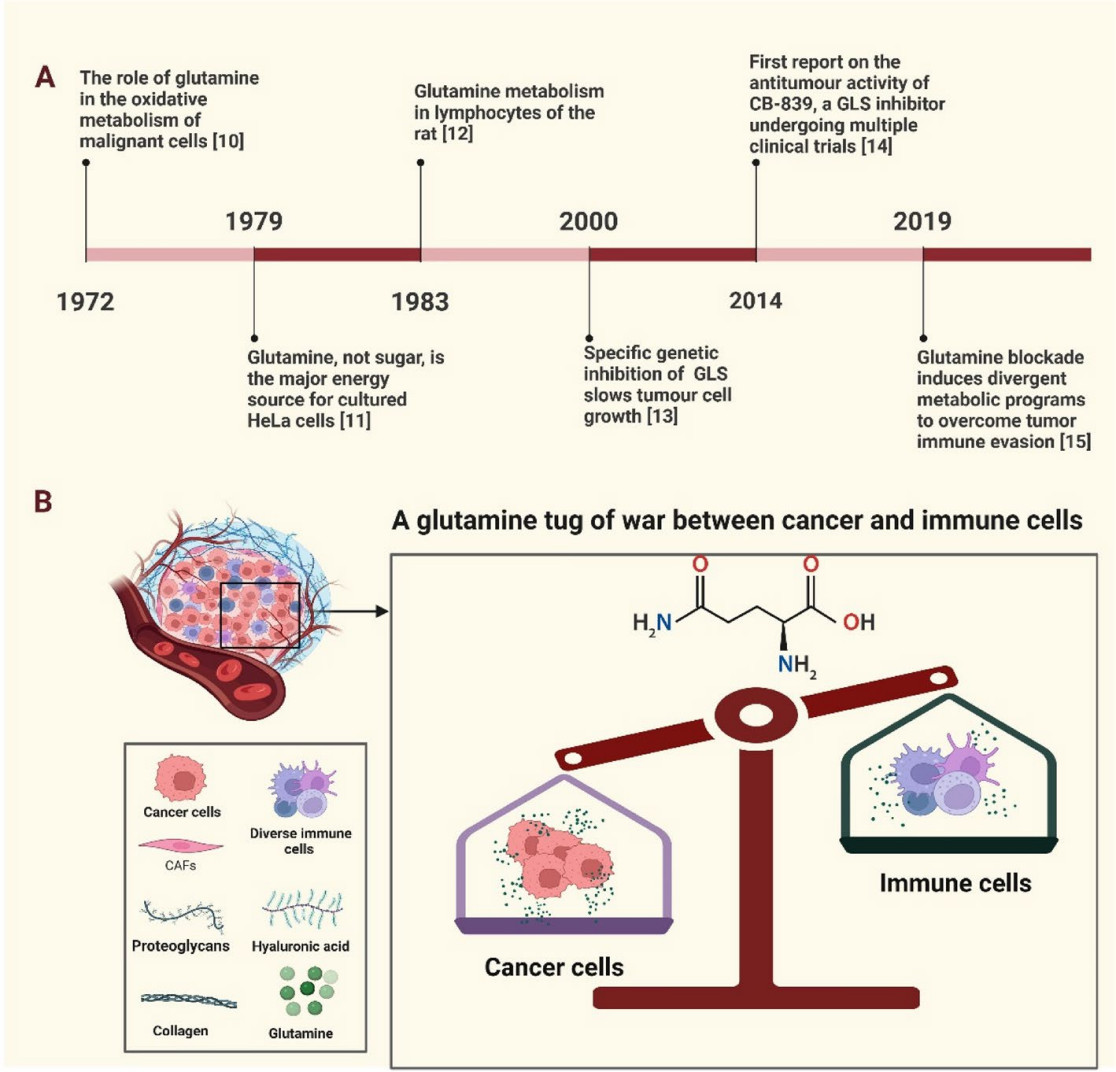
Glutamine emerges as a rising star in the field of cancer immunotherapy. **A** The timeline of major breakthroughs in glutamine metabolism. **B** The dynamic interplay between cancer cells and immune cells in their competition for the limited pool of available glutamine. GLS, glutaminase

Glutamine, traditionally perceived as a non-essential amino acid, has emerged as a key player in tumor biology [8]. Initially identified by Schulze in 1883, with its synthesis elucidated by Hans Krebs in 1935, glutamine's significance in organismal homeostasis was subsequently investigated through animal experiments [9]. Figure 1A depicts the significant progress made over the past decades in unraveling the complexities of cancer and immune cell metabolism. In 1972, the crucial role of glutamine in malignant cell oxidative metabolism was discovered [10]. Subsequently, in 1979, the dependence of cultured HeLa cells on glutamine as their primary energy source was revealed, further emphasizing its critical role in maintaining cancer cell metabolism [11]. Advances in 1983 further deepened our understanding of glutamine's involvement in lymphocyte metabolism [12]. By the year 2000, specific gene suppression of glutaminase (GLS) became a promising strategy for effectively impeding tumor cell growth [13]. In 2014, the GLS inhibitor CB-839, undergoing clinical trials, was reported to possess potent anti-tumor activity, offering a novel avenue for targeted cancer therapy through modulation of glutamine metabolism [14]. In the course of persistent investigation, researchers in 2019 made a striking observation, unveiling disparate metabolic traits exhibited by cancer cells and immune cells. Furthermore, the revelation that impeding the glutamine metabolism of cancer cells could enhance the immune microenvironment and bolster the effectiveness of immunotherapy has provided a compelling impetus for further exploration [15]. This groundbreaking finding has sparked a fervent wave of inquiry into the intricate interplay between glutamine metabolism and the immune landscape of cancer, captivating scientists and propelling them towards further exploration in this captivating realm of research.

As the field continues to advance in recent years, researchers have discovered that cancer cells exhibit a distinct metabolic profile characterized by accelerated rates of proliferation and high energy demands [8]. To meet these requirements, cancer cells reprogram their metabolic pathways to ensure an adequate supply of nutrients and energy sources [16]. Glutamine emerges as a significant player in this metabolic reprogramming, as it provides carbon and nitrogen for macromolecule synthesis, including proteins, nucleotides, and lipids essential for cancer cell growth and division [17]. Similarly, immune cells such as T cells, natural killer (NK) cells, and macrophages require energy to mount effective immune responses against cancer cells [18, 19]. However, within the TME, the availability of nutrients, including glutamine, is limited due to competition among diverse cell populations [20]. Figure 1B shows the metabolic competition creates a tug-of-war between cancer and immune cells, as they vie for access to the limited glutamine resources [21, 22]. In light of the intricate metabolic competition between cancer and immune cells for limited glutamine resources, understanding the molecular mechanisms underlying this "tug-of-war" becomes crucial. Emerging evidence suggests that cancer cells utilize various mechanisms to gain a competitive advantage over immune cells in acquiring and utilizing glutamine [7, 23]. On the other hand, immune cells face the challenge of acquiring enough glutamine to maintain their effector functions and combat the tumor [7]. The aforementioned evidence suggests that the involvement of glutamine in tumor biology positions it as a pivotal player in the fight against cancer. A comprehensive understanding of the molecular mechanisms underlying glutamine metabolism and its impact on both cancer cells and immune cells holds great promise in elucidating immune evasion mechanisms and facilitating the development of effective anti-cancer treatment strategies.

## Glutamine metabolism in cancer cells

### Glutamine addiction in cancer cells

Uncontrolled cell proliferation represents a prevailing characteristic of tumorigenesis, necessitating not only a consistent provision of essential nutrients and metabolites but also necessitating adaptations in energy metabolism to sustain cellular growth and division [24]. Glutamine, an indispensable nutrient for rapidly dividing cells like tumor cells, can be obtained from the diet or synthesized de novo via the enzymatic activity of glutamine synthase [17]. Furthermore, during periods of nutrient deprivation, glutamine acquisition can occur through the autophagic degradation of macromolecules [25]. Most cancers exhibit a strong affinity for glutamine, and several cancer cell lines, such as glioblastoma, heavily rely on this amino acid for their survival, as they are unable to sustain themselves without an external source of supply [26]. The dependency of transformed cells on glutamine for their metabolic processes is now recognized as the distinct phenomenon known as glutamine addiction [27]. Glutamine is an essential component for sustaining the survival and proliferation of cancer cells, providing fuel for diverse metabolic pathways such as the Krebs cycle, redox homeostasis, and the synthesis of crucial cellular building blocks including nucleic acids, fatty acids, glutathione (GSH), and other amino acids [28].

**Fig. 2:**
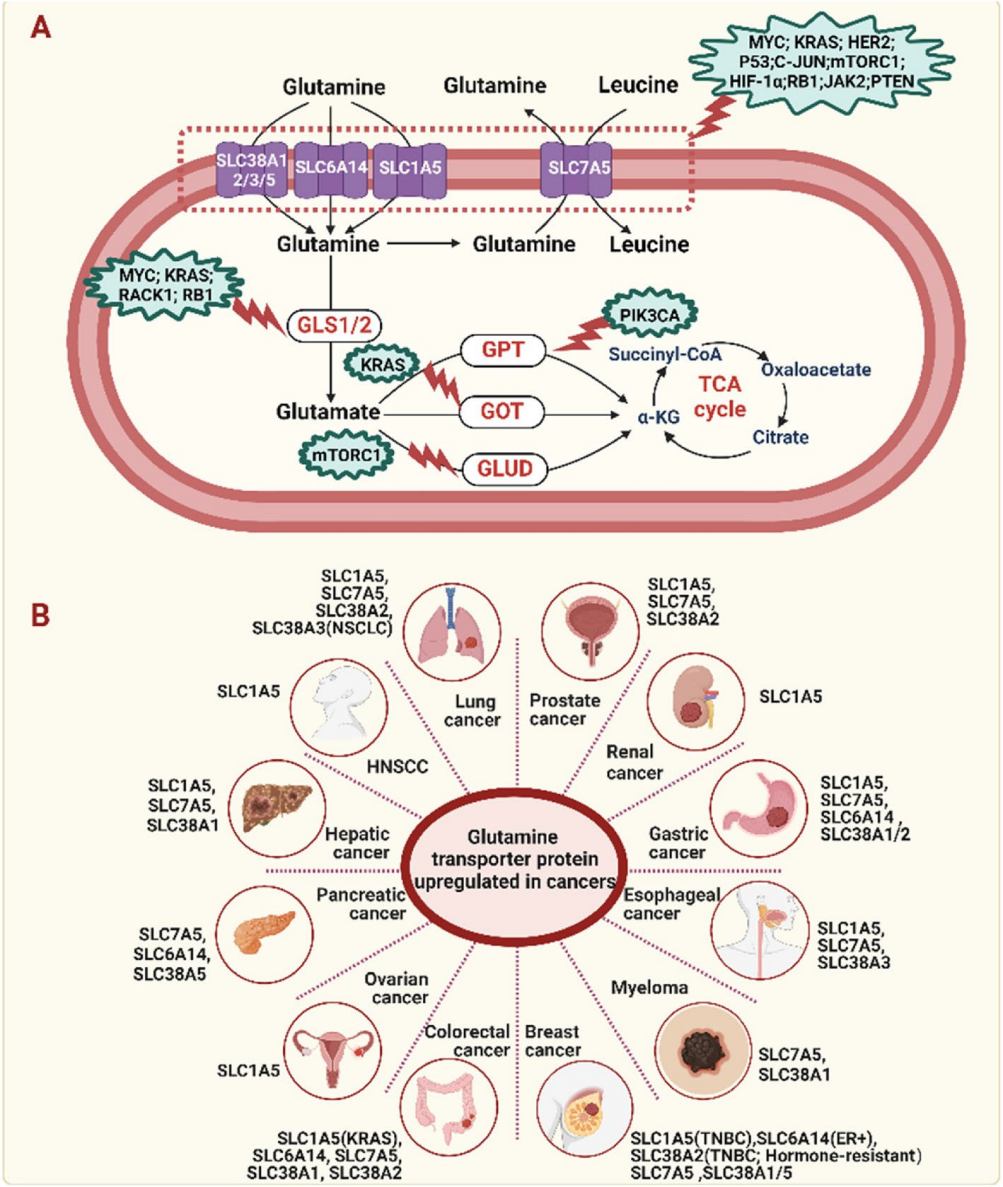
Glutamine addiction in cancer cells. **A** The process of glutamine catabolism in tumor cells, as well as potential gene alterations that can impact glutamine metabolism. Glutamine is transported into the cell through membrane amino acid transporters, such as SLC1A5, SLC6A14, and SLC38A1/2, where it undergoes a dehydrogenation reaction catalyzed by GLS to form glutamate. Glutamate then converts to α-KG through the action of GLUD, GOT or GPT, entering the TCA cycle for energy production. The green region represents the influence of various gene alterations on different nodes of glutamine metabolism. **B** Multiple glutamine transporter proteins have been observed to undergo upregulation across different types of cancer. GLS, glutaminase; GPT, glutamate pyruvate transaminase; GOT, glutamic-oxaloacetic transaminase; GLUD, glutamate dehydrogenase; α-KG: α-ketoglutarate; TCA: tricarboxylic acid; TNBC: triple-negative breast cancer; NSCLC: non-small cell lung cancer; HNSCC: head and neck squamous cell carcinoma; ER + : estrogen receptor positive

Figure 2A shows the process of glutamine catabolism in tumor cells. The first step in glutamine metabolism requires entry into the interior of tumor cells. Glutamine is hydrophilic and therefore cannot traverse the plasma membrane without the assistance of selective transport proteins [29]. Given the high demand of tumor cells for glutamine, it can be reasonably inferred that certain selective amino acids must be upregulated in tumor cells to meet this requirement. Figure 2B summarizes the currently identified glutamine transport proteins in tumor cells that play a pivotal role in tumor glutamine metabolism. SLC1A5 (ASCT2) is a vital transporter for neutral amino acids, relying on sodium to facilitate exchanges with asparagine, serine, or threonine [30]. Its exceptional affinity for glutamine, especially in acidic environments, enables efficient transport of this amino acid into cancer cells thriving under such conditions [31]. SLC1A5 is highly expressed in diverse solid tumors, including triple-negative breast cancer (TNBC) [32], KRAS-mutated colorectal cancer (CRC) [33], esophageal cancer [30], gastric cancer [34], lung cancer [35], ovarian cancer [36], prostate cancer [37], renal cell [38], head and neck squamous cell carcinoma (HNSCC) [39] and hepatocellular carcinoma [40]. SLC7A5, also known as L-amino acid transporter 1(LAT1), functions differently [41]. It is not an active transport protein that relies on transmembrane ion gradients [29]. On the contrary, it acts as a compulsory exchanger, mediating the influx of leucine into the cell and the efflux of glutamine out of the cell [29, 42]. Hence, the question arises: How does SLC7A5, as a mandatory exchanger, facilitate the entry of leucine into the cell? Extensive exploration has revealed that the exchange mechanism between SLC1A5 and SLC7A5 plays a role in balancing cytoplasmic amino acid pools [43]. Specifically, in the functional coupling between SLC7A5 and SLC1A5, glutamine enters cancer cells through SLC1A5, which then effluxes out of the cells via SLC7A5, linked to the entry of leucine [44]. Consistent with this, increased expression levels of SLC7A5 have been observed in various types of cancer such as breast cancer [45], KRAS-mutated CRC [46], gastric cancer [47], lung cancer [48], pancreatic cancer [49], prostate cancer [50], melanoma [51], ovarian cancer [52] and hepatocellular carcinomas [53], and esophageal cancer [54].

In addition to SLC1A5 and SLC7A5, there are other transport proteins that contribute to glutamine metabolism in tumor cells (Fig. 2B). SLC6A14, also known as the amino acid transporter B^0,+^ (ATB^0,+^), maintains a unidirectional influx of glutamine across the transmembrane gradient, concurrently binding with 2 Na + ions and 1 Cl- [55, 56]. SLC6A14 exhibits elevated expression in CRC [57], estrogen receptor positive (ER +) breast cancer [58], and pancreatic cancer [59], concomitant with their proliferative capacity. Additionally, upregulation of SLC6A14 expression has been observed in gastric cancer, and depletion of SLC6A14 leads to the suppression of epithelial-mesenchymal transition-induced metastasis in gastric cancer through disruption of the PI3K/Akt/mTORC1 pathway [60]. Furthermore, SLC38 family transporters have been identified as key players in glutamine transport in cancer [61]. These transporters can be classified into two distinct subfamilies characterized by their unique operational features: system A, encompassing prominent members SLC38A1(SNAT1) and SLC38A2 (SNAT2), and system N, encompassing transporters like SLC38A3 (SNAT3), and SLC38A5 (SNAT5) [61]. SLC38A1 exhibits upregulated expression in breast cancer [62], gastric cancer [63], CRC [64], lung cancer [65], hepatocellular carcinoma [66] and melanoma [67], displaying a strong correlation with enhanced proliferation. In prostate cancer [68] and lung cancer [69], SLC38A2 is markedly expressed, thereby playing a significant role in tumorigenesis. Furthermore, a distinct expression profile of SLC38A2 emerges across breast cancer subtypes when compared to normal tissue. Data derived from the TCGA database illustrate a trend towards decreased SLC38A2 expression in breast cancer relative to adjacent normal tissues, but these did not reach statistical significance [70]. Notably, while SLC38A2 is downregulated in ductal breast carcinoma, an upregulation is observed in TNBC [71] and hormone-resistant breast cancers [72]. In TNBC, SLC38A2 plays a crucial role in promoting glutamine dependency and conferring resistance to oxidative stress [71]. As a glutamine transporter induced by oxygen deprivation in breast cancer, the upregulation of SLC38A2 results in resistance to hormone therapy and subsequently a poorer prognosis [72]. These diverse observations highlight potential variations in the involvement of SLC38A2 across different pathological subtypes of breast cancer and at various treatment stages, underscoring the need to consider the heterogeneity when targeting SLC38A2 for therapeutic interventions in breast cancer. As another pivotal l-glutamine transporter, SLC38A3 demonstrates marked upregulation in metastatic NSCLC cells, with its expression levels intricately associated with the prognostic outcomes of NSCLC patients [73]. SLC38A5 is an Na + /H + exchanger that relies on amino acids, including glutamine, to enable the transport of Na + and amino acids into cells, while concurrently promoting H + efflux [74]. In pancreatic [75] and breast cancer [76] cells, upregulation of SLC38A5 promotes cell proliferation, while its knockdown significantly reduces cell growth impairs glutamine uptake. The unveiling of SLC1A5, SLC7A5, SLC6A14, and the SLC38 family transporters, along with their distinctive functional characteristics, enriches our comprehension of the intricate nature of glutamine transport in cancer. These discoveries also present exciting prospects for the development of groundbreaking therapies tailored to selectively target these pivotal transport proteins.

Upon entry into the cell through the aforementioned glutamine transporters, glutamine is enzymatically deaminated by GLS, leading to the generation of glutamate (Fig. 2A). It then further converts to α-ketoglutarate (α-KG) through the action of glutamate dehydrogenase (GLUD) or transaminases, entering the tricarboxylic acid (TCA) cycle, generating reduced nicotinamide adenine dinucleotide (NADH) and flavin adenine dinucleotide (FADH2), and ultimately producing adenosine triphosphate (ATP). The nitrogen derived from glutamine in these processes plays a role in the synthesis of nucleotide precursors [77]. The release of ammonia from GLS and GLUD plays a crucial role in the synthesis of carbamoyl phosphate in the urea cycle. Carbamoyl phosphate synthetase I (CPSI) facilitate the incorporation of ammonia into carbamoyl phosphate. In contrast, carbamoyl phosphate synthetase II (CPSII) utilizes the amide group from glutamine to generate carbamoyl phosphate, which is an important step in pyrimidine synthesis. Cytidine triphosphate synthetase (CTPS) converts uridine triphosphate (UTP) into CTP by utilizing the amide group derived from glutamine. In the process of purine synthesis, 5-phosphoribosyl-α-pyrophosphate (PRPP) generated from the pentose phosphate pathway (PPP) is converted to phosphoribosyl-β-amine (PRA) through the action of phosphoribosyl pyrophosphate amidotransferase (PPAT) when an amide group from glutamine is introduced. Moreover, the amide group from glutamine can also be transferred to formylglycinamide ribonucleotide (FGAR), which subsequently forms phosphoribosyl-formylglycinamidine (FGAM) through the catalytic activity of 5'-phosphoribosylformylglycinamidine synthase (PFAS) during nucleotide synthesis. These investigations highlight the critical role of glutamine-derived nitrogen in nucleotide precursor synthesis and emphasize the significance of glutamine as a rate-limiting factor in cancer cell proliferation. A study observed that exogenous glutamine can synthesize nucleotides in human primary cancer samples cultured in vitro [78]. Cancer cells lacking glutamine experience cell cycle arrest, emphasizing the importance of glutamine as a nitrogen source [79]. Glutamine metabolism can also serve as an alternative carbon source for the TCA cycle, promoting fatty acid synthesis through reductive carboxylation reactions [80]. Researchers also observed that approximately half of the nonessential amino acids required for protein synthesis in tumor cells are derived from glutamine using isotopic tracing methods [77]. Additionally, glutamine is crucial for maintaining cellular redox homeostasis. It regulates the level of reactive oxygen species (ROS) by controlling the synthesis of GSH. Glutathione, a tripeptide (Glu-Cys-Gly), is involved in neutralizing peroxides [81]. Overall, glutamine plays an indispensable role in fueling cancer cell proliferation and biosynthesis, further understanding of the complexities of glutamine metabolism in tumors may provide novel insights into the development of more effective cancer treatments.

### Gene alterations modulating glutamine dependency in cancer

The dependency of glutamine metabolism in cancer is not simply a passive adaptation to proliferative state, but rather a tightly regulated response to a multitude of intracellular and extracellular factors, including potential genetic alterations. To date, the extent of glutamine dependency in cancer cells has been found to be modulated by oncogenic or tumor suppressor gene alterations, which are summarized in Fig. 2A. MYC is the third most commonly oncogene in human cancer [82]. Yuneva et al. were the first to observe its ability to drive proliferation of cells dependent on glutamine [83]. Further research revealed that MYC actively stimulates gene expression related to glutamine metabolism. Specifically, MYC upregulates the glutamine transporter and induces the expression of GLS at both mRNA and protein levels [84, 85]. In glioblastoma cells, the sensitivity to glutamine deprivation depends on c-MYC and can be inhibited by targeting MYC expression [85]. This dependency is not limited to MYC-driven tumor cells but also occurs in various other cancer cell lines, including lymphomas, prostate cancer, osteosarcoma, renal cell carcinoma, colorectal cancer, and small cell lung cancer [84, 86–90].These tumor cells rely on glutamine for cell survival and growth when MYC is activated. Interestingly, glutamine itself can also regulate the expression of c-MYC protein in some tumor cells. In addition to MYC, mutations in the key gene KRAS, which are associated with c-MYC protein stability and activity, similarly result in dependence on exogenous glutamine for cell growth and proliferation. Recent studies have revealed that cancer cells with KRAS mutations utilize amino acids such as glutamine and leucine to accelerate energy metabolism and redox balance through GSH synthesis and macromolecule biosynthesis. This mutation leads to upregulation of specific amino acid transporters (AATs) in cancer cells, such as SLC7A5 and SLC38A2, which are associated with enhanced amino acid uptake. Knocking down KRAS reduces the absorption of amino acids by colorectal cancer cells, while overexpression of mutated KRAS increases the expression levels of AATs [91]. Similarly, it has been observed that KRAS mutation induces activation of the glutamine metabolism pathway in pancreatic cancer by enhancing the expression of pivotal enzymes responsible for glutamine degradation [92]. For instance, glutamic-oxaloacetic transaminase (GOT) 2 is a key component of mutant KRAS-mediated rewiring of glutamine metabolism in pancreatic ductal adenocarcinoma [6]. Additionally, higher mRNA expression of GLS has been detected in KRAS-mutated non-small cell lung cancer (NSCLC) [93].

The protein kinase, mechanistic target of rapamycin (mTOR), orchestrates cellular processes via two distinct complexes, mTORC1 and mTORC2 [94]. Notably, the mTORC1 pathway exhibits persistent hyperactivation in cancer, highlighting the need for deeper investigation into its oncogenic implications [95]. Interestingly, sufficient levels of glutamine have been shown to activate the mTORC1 pathway, and reciprocally, mTORC1 regulates glutamine metabolism [96]. It has been demonstrated that the activation of the mTORC1 pathway is associated with cancer cells' dependence on glutamine [97]. In human cancer epithelial cell lines, such as colorectal and prostate cancer cells, mTORC1 promotes the utilization of glutamine by activating GLUD [98]. Furthermore, mTORC1 not only regulates GLUD, but also actively controls GLS through S6K1-dependent c-MYC, enhancing cancer cells' uptake of glutamine [99]. Recently, Chen et al. uncovered that the loss of receptor for activated C kinase 1 (RACK1) elicits activation of the AKT/mTOR signaling pathway, upregulating SLC1A5 and fostering glutamine addiction in gastric cancer cells [100]. PIK3CA serves as a crucial regulator in the context of glutamine addiction. Previous studies have revealed that PIK3CA mutations reprogram glutamine metabolism by upregulating the expression of glutamate pyruvate transaminase (GPT) 2, thereby increasing glutamine dependency in CRC cells [101]. Recent investigations have reported that PIK3CA mutations modulate SIRT4 expression through negative regulation of the epigenetic factor EP300, ultimately influencing GPT1 and altering glutamine metabolism in cancer [99]. The activated ErbB2 (also known as neu or HER2) is a major driver of cancer [102]. In ErbB2-transformed MCF10A cells, there is increased expression of GLS mRNA and protein compared to MCF-10A cells, and knockdown of ErbB2 leads to downregulation of GLS expression [103]. Further investigations have revealed that activated ErbB2 stimulates the expression of GLS in cancer cells through the PI3K/Akt-independent nuclear factor-kappa B (NF-kB) pathway in breast and gastric cancer [103, 104]. Previous investigations have provided evidence demonstrating the pivotal role of p53 in governing glutamine metabolism to uphold the redox equilibrium of tumor cells [105]. Upon activation, p53 orchestrates the upregulation of GLS2 expression, thereby facilitating the breakdown of glutamine and heightening the intracellular levels of GSH [106].

In addition, there are several other genetic alterations associated with the glutamine dependency of cancer, and these mutations in genes may influence the extent to which tumor cells rely on glutaminase metabolism. In mouse murine pro-lymphoid (BaF3) cells with mutated JAK2 displayed significantly enhanced glutaminase metabolism and increased expression of GLS compared to wild-type cells [107]. Furthermore, the oncogenic transcription factor c-JUN was found to regulate the gene expression of GLS [108]. Hypoxic conditions induced the reductive metabolism of glutaminase in tumor cells, resulting in the transcriptional activation of GLS via HIF-1α upregulation [90]. The increased dependence on glutaminase metabolism is associated with mutations in BRAF in melanoma cells [109]. Loss of the PTEN gene has been found to decrease ubiquitination of GLS, potentially leading to an increase in intracellular levels of glutamine [110]. Conversely, loss of the RB1 gene upregulates the expression of GLS and SLC1A5 genes, further promoting cellular uptake and utilization of glutamine [111]. Furthermore, the upregulation of transforming growth factor (TGF)-β and WNT signaling pathways promotes the activation of SNAIL and DLX2, which in turn upregulate the expression of GLS [112]. These findings provide important clues for further investigation into the metabolic remodeling of tumor cells and potential targeted therapies.

## Glutamine metabolism in immune cells

### Crucial role of glutamine in immune cell

**Fig. 3:**
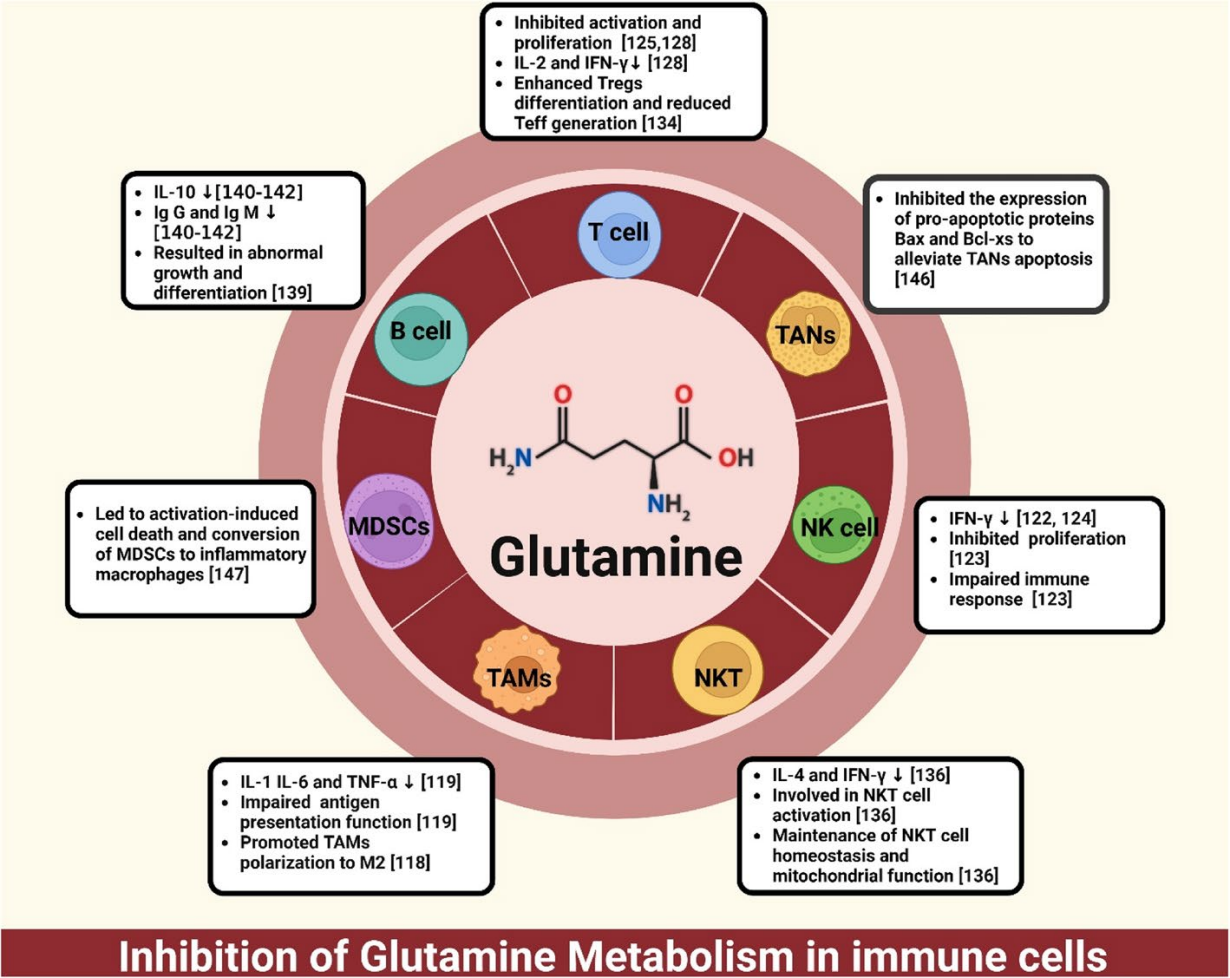
The impact of glutamine metabolism inhibition on diverse immune cell populations. Created with BioRender.com. TANs, tumor-associated neutrophils; TAMs, tumor-associated macrophages; NK, natural killer; MDSCs: myeloid-derived suppressive cells; IFN, interferons; IL, interleukin; TNF: tumor necrosis factor

The early-to-mid 1980s witnessed a significant breakthrough in the field of immunology, as researchers at Eric Newsholme's laboratory revealed the remarkable utilization of glutamine by immune cells, including lymphocytes and macrophages, alongside glucose [113]. Glutamine, as a fundamental fuel in immune cell metabolism, plays a pivotal role in the functionality and activation of immune cells. It has been observed that lymphocytes exhibit remarkable utilization rates of approximately 2.7 μmol/minute per gram dry weight under aerobic conditions [12]. Not only that, stimulation with concanavalin A leads to a substantial 51% augmentation in glutamine utilization [12]. In the face of restricted glucose availability, immune cells exhibit a profound dependence on the uptake and catabolism of glutamine as a means to sustain the TCA cycle, a pivotal process crucial for their ongoing viability and functionality [114–116]. This discovery propelled the exploration of the critical role played by glutamine in the functioning and activation of immune cells. Consequently, glutamine metabolism has emerged as a pivotal regulator in maintaining immune cell homeostasis. Table 1 summarizes the biological functions in which several AATs have been identified as critical mediators of glutamine uptake in immune cells. Figure 3 demonstrates how inhibiting glutamine metabolism affects a range of immune cell populations. In-depth understanding of these mechanisms holds immense potential for advancing our comprehension of immunology and revolutionizing cancer therapeutic interventions.

**Table 1:** Membrane-bound transporters for glutamine amino acid, their characteristics, and the immune cells with upregulated expression

**Immune cell**	**Glutamine transporters**	**Biological functions**	**Refs**
**NK cell**	SLC1A5	Upregulation of SLC1A5 expression enhances IFN-γ production in NK cells	[117]
**T cell**	SLC1A5	SLC1A5 is essential for the rapid uptake of glutamine during the activation of naive T cells	[118]
	SLC1A5	T cells lacking SLC1A5 exhibit significantly reduced glutamine uptake and mTORC1 activation upon TCR engagement, leading to blocked differentiation of Th1 and Th17 cells	[119]
	SLC6A14	SLC6A14 serves as a compensatory glutamine transporter in CD8 + T cells, allowing them to maintain effector function and sustain glutamine uptake even when the primary transporter is inhibited, distinguishing them from triple-negative breast cancer cells which are sensitive to glutamine uptake inhibition	[119]
	SLC38A2	SLC38A2-mediated glutamine transport can reprogram the generation and persistence of memory T cells	[120]
**B cell**	SLC1A5	Inhibiting the glutamine transporter SLC1A5 resulted in decreased IgM production in B cells	[121]
**DC cell**	SLC38A2	SLC38A2 expressed by DCs is essential for maintaining the effector function of antigen-specific CD8 + T cells in the TME, without affecting antigen uptake or migratory capacity of DCs	[7]

*NK* Natural killer, *DC* Dendritic cell

### Glutamine metabolism in macrophages and NK cells

Tumor-associated macrophages (TAMs), including both M1 and M2 subtypes, are among the most abundant cells in the tumor microenvironment [122]. M1 macrophages actively participate in immune responses, executing immune surveillance through the secretion of inflammatory cytokines and chemokines, and engaging in professional antigen presentation [122]. Conversely, M2 macrophages demonstrate diminished antigen-presenting capacity, releasing anti-inflammatory cytokines like interleukin (IL)-10 or TGF-β, and suppressing immune responses [122]. TAMs adjust their activation state, synthesis of inflammatory cytokines, and proliferation of peroxisomes by modulating glutamine metabolism [123]. The crucial role of glutamine in macrophages has been emphasized through early animal experiments, which have unveiled its irreplaceable involvement in cytokine generation, including prominent players like IL-1, IL-6, and tumour necrosis factor (TNF)-α [124]. Additionally, glutamine has been shown to contribute indispensably to antigen presentation and phagocytic function, further emphasizing its critical significance in macrophage biology [124]. The orchestration of glutamine utilization stands out as a pivotal metabolic factor governing macrophage reprogramming and phenotypic polarization [123, 125]. It was reported that the breakdown of glutamine generates α-KG, which plays a crucial role in selectively activating M2 macrophages [123]. Glutamine can also impact the metabolism of other nutrients, influencing the metabolic function of macrophages [126]. Recent research indicates that glutamine usage reinforces fatty acid oxidation -induced pro-inflammatory and anti-tumorigenic activation by fine-tuning the nicotinamide adenine dinucleotide (NAD) + /NADH ratio via glutamine-to-lactate conversion [126]. These findings not only deepen our understanding of the metabolic regulation of macrophages in the TME but also provide a novel research perspective for exploring the role of glutamine in NK cells. Initial investigations have underscored the pivotal role of glutamine in NK cells, revealing that the upregulation of SLC1A5 significantly boosts interferon (IFN)-γ production by enhancing glutamine uptake [117]. Subsequent studies have further illuminated the critical importance of glutamine availability, showing that a reduction in c-MYC protein levels, which leads to diminished cellular proliferation and impaired NK cell function, results primarily from glutamine scarcity rather than the direct inhibition of glutaminolysis [127]. Building on these revelations, recent research has emphasized the metabolic adaptability and resilience of NK cells, particularly highlighting their reliance on glutamine alongside other substrates such as fatty acids or acetate, to precisely regulate IFN-γ expression in response to physiological needs [128]. This nuanced appreciation of glutamine's central role in modulating the metabolic pathways crucial for immune cell efficacy opens new avenues for therapeutic intervention.

### Glutamine metabolism in T cells and B cells

The metabolic characteristics of T cells are markedly shaped by their activation status [129]. Quiescent T cells exhibit reduced glycolysis rates and diminished glutamine metabolism to support crucial biosynthetic pathways essential for survival [129]. In contrast, activated T effector cells display heightened glycolysis and intensified glutamine metabolism, facilitating the protein and nucleotide demands of rapidly dividing T cells [129]. Consistent with this observation, the process of T cell activation is accompanied by the upregulation of both SLC1A5 and SLC38A1, leading to a notable increase in glutamine uptake [130]. Previous research has found that glutamine, through breakdown, supplements the oxidative phosphorylation (OXPHOS) cascade reaction in T cells and promotes the synthesis of lipids, polyamines, and amino acids [118]. Lowering glutamine levels to 50% of the standard culture concentration significantly inhibits mouse T cell proliferation, while reducing it to below 10% completely blocks proliferation [131]. Similarly, treatment with the glutamine antagonist JHU083 results in decreased T cell activation and proliferation [132]. Complete removal of glutamine from the culture medium hinders cell growth and the secretion of IL-2 and IFN-γ by activated mouse splenic T cells [131]. Furthermore, activation of memory CD8 + T cells through CD8 antibody-mediated stimulation triggers downstream signaling of the T cell receptor, enhancing T cell-mediated cytotoxicity and promoting the production of effector cytokines, which is dependent on glucose and glutamine [133]. This finding further highlights the critical role of glutamine in regulating T cell function and immune response.

Recent studies also shed light on its critical involvement in maintaining the equilibrium among distinct T cell subpopulations. The role of glutamine in T cells is mediated through rapid uptake facilitated by SLC1A5, promoting the activation of naïve T cells and regulating T cell immune response [119]. Deficiency of SLC1A5 impacts the induction of Th1 and Th17 cells, weakening the inflammatory T cell response [119]. In CD8 + T cells, SLC6A14 assumes the role of a glutamine transporter, particularly under conditions where the primary transport mechanism is inhibited, safeguarding effector functionality and continuous glutamine absorption [134]. Moreover, the role of SLC38A2 in mediating glutamine transport contributes to the reprogramming of the emergence and maintenance of memory T cells, highlighting the impact of glutamine availability on T cell memory [120]. Furthermore, prior investigations have suggested that a shortage of glutamine hinders the proliferation of Teff cells and the production of cytokines, while promoting the expansion of regulatory T cells (Tregs) population [135]. Multiple metabolic pathways associated with glutamine, such as glutamine-mTOR signaling, glutamine-glutamate-other amino acid pathway, glutamine-ISR stress pathway, and glutamine-autophagy pathway, have been observed to modulate the differentiation of Th17 and Tregs [136]. Glutamine plays a pivotal role in the orchestration of natural killer T (NKT) cell metabolism. In the quiescent state, NKT cells exhibit elevated levels of glutamine compared to CD4 + T cells, and upon activation, they demonstrate enhanced glutamine catabolism [137]. Furthermore, glutamine metabolism seems to be critical for NKT cell homeostasis and mitochondrial functions. Activated NKT cells utilize glutamine to fuel the tricarboxylic acid cycle and GSH synthesis, with nitrogen derived from glutamine driving protein glycosylation through the hexosamine biosynthetic pathway [137]. This heightened glutamine metabolism and the hexosamine biosynthesis pathway distinctly regulate the production of IL-4 and IFN-γ [137]. These findings underscore the vital role of glutamine in promoting effective immune cell responses and suggest that interventions aimed at supplementing glutamine levels may enhance T cell activation and bolster anti-tumor immunity.

The significance of glutamine extends beyond T lymphocytes, as it also plays a pivotal role in the metabolism of B lymphocytes. It has been observed that the differentiation of B cells into plasma cells and memory B cells depends on the availability of an adequate supply of glutamine [138]. Anne Le and colleagues have revealed a glucose-independent TCA cycle, which is exclusively fueled by glutamine metabolism in B cells [139]. Remarkably, this metabolic route remains functional even in the presence of abundant glucose and exhibits sustained activity under hypoxic conditions [139]. Waters et al.'s findings indicate that glutamine constraint jeopardizes both the growth and differentiation of B cells [140]. Moreover, the production of antibodies and cytokines by B cells is dependent on the catabolism of glutamine. Inhibition of GLS expression results in reduced immunoglobulin (Ig) G and IgM antibody production, and glutaminase blockade decreased downstream mTOR activation and attenuated IL-10 secretion [121, 141, 142]. Notably, SLC1A5, a critical glutamine transporter, plays a pivotal role in regulating immunoglobulin synthesis in B cells. Its inhibition leads to a significant reduction in IgM production, highlighting the crucial role of SLC1A5 as a transportation protein in orchestrating the intricate process of immunoglobulin generation within B cells [121]. The significance of comprehending the pivotal role of glutamine metabolism and its transporters in regulating B cell activity is further underscored by these compelling findings. Importantly, this research also highlights the criticality of investigating glutamine metabolism within the context of tumor immunity. Such investigations hold great promise for unraveling novel therapeutic strategies aimed at harnessing the power of glutamine metabolism to combat cancer.

### Glutamine in neutrophils and myeloid-derived suppressor cells (MDSCs)

The pivotal role of glutamine metabolism extends to neutrophils and myeloid cells. In recent studies have shown that glutamine also plays a vital role in maintaining the survival and function of immune cells such as neutrophils. The allure of tumor-released chemotactic factors beckons neutrophils to the tumor site, where tumor-associated neutrophils (TANs) can bolster CD8 + T cell responses and anti-tumor activity in the absence of tumor-derived TGF-β. However, in the presence of TGF-β, TANs fuel the pro-tumor activity of CD8 + T cells [143]. Notably, when compared to other white blood cells such as macrophages and lymphocytes, neutrophils exhibit the highest rate of glutamine consumption [143–145]. These cells exhibit a remarkably high utilization rate of glutamine, which is associated with a significant reduction in their apoptosis rate. Intriguingly, glutamine has been shown to mitigate neutrophil apoptosis by suppressing the expression of pro-apoptotic proteins Bax and Bcl-xs, as supported by compelling evidence [146]. Furthermore, by targeting the glutamine metabolism of MDSCs, an exciting breakthrough was achieved. Inhibiting the glutamine metabolism of MDSCs not only triggered activation-induced cell death but also facilitated their transformation into pro-inflammatory macrophages [147]. These findings highlight the complex interplay between glutamine metabolism and immune cell homeostasis, underscoring the critical importance of maintaining optimal glutamine levels for both tumor progression and immune function.

## The impact of glutamine metabolic competition on the tumor microenvironment

### Tumor-induced glutamine competition leads to reduced uptake in immune cells

**Fig. 4:**
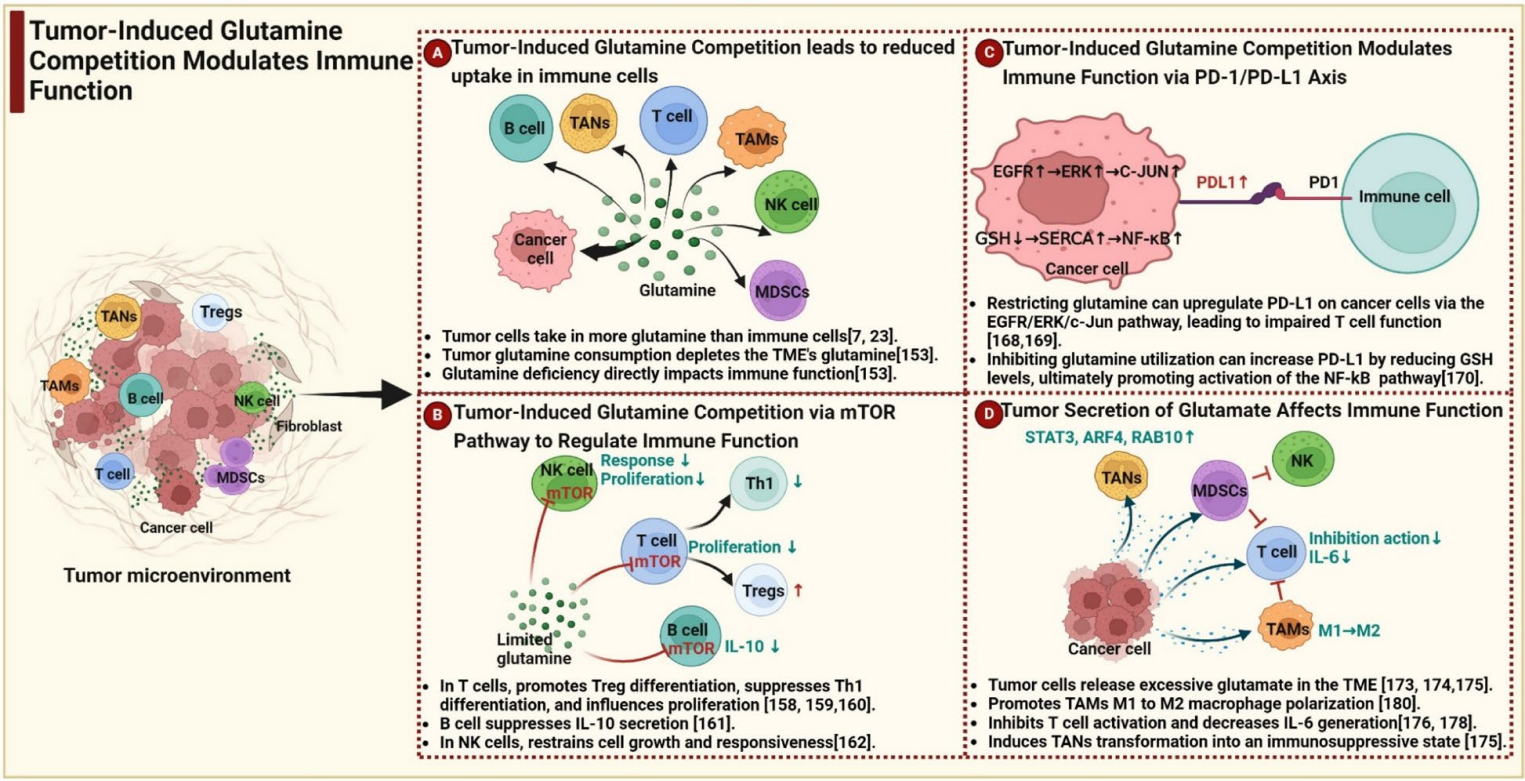
Tumor-induced glutamine metabolic competition shapes immune cell responses in the tumor microenvironment. **A** Tumor-induced glutamine competition leads to reduced uptake in immune cells. **B** Tumor-induced glutamine competition via mTOR pathway to regulate immune function. **C** Tumor-induced glutamine competition via the PD-1/PD-L1 axis to regulate immune function. **D** Tumor-induced glutamine competition promotes glutamate secretion to modulate immune responses. Red solid T arrows direct inhibitory interactions. Created with BioRender.com. TANs, tumor-associated neutrophils; TAMs, tumor-associated macrophages; NK, natural killer; Tregs, regulatory T cells; MDSCs, myeloid-derived suppressive cells; PD-1, programmed cell death 1; PD-L1, programmed cell death-ligand 1; IFN, interferons; IL, interleukin; TME, tumor microenvironment

Figure 4A visually demonstrates the differential uptake of glutamine between immune cells and tumor cells in the TME. Tumor cells activate multiple carcinogenic pathways, leading to increased expression of transporters and metabolism-related enzymes, which in turn results in enhanced uptake of glutamine by tumor cells and massive consumption of glutamine in the TME [7, 23]. As evidenced by the remarkable 18F-Gln tracing results, tumor cells exhibit the highest capacity for glutamine uptake, thus establishing their dominance in the competition for this critical resource [148]. The immune cells have a certain demand for glutamine, and a decrease in glutamine levels can threaten the integrity and function of immune cells [149]. Unfortunately, compared to tumor cells, immune cells have difficulty adapting to the constantly evolving tumor through "microevolution" because the latter develops new immune escape mechanisms [150]. As observed at the gene level tumor cells have significantly higher scores for glutamine metabolism genes than CD8 + T cells, highlighting their metabolic differences with immune cells [151]. Notably, supplementing glutamine in the tumor microenvironment led to a slowdown in tumor growth and restored T cell infiltration, function, and memory phenotype [152]. The latest research further supports the above view, with a co-culture model constructed to simulate the interaction between breast cancer spheroids and Jurkat T cells [153]. They found that the high energy demand of breast cancer cells leads to glutamine depletion, thereby affecting T cell function [153]. In a recent study, researchers have uncovered that immunologically-hot melanoma exhibits a heightened reliance on glutamine compared to immunologically-cold melanoma, both in vivo and in vitro [154]. A lack of amino acids such as glutamine not only damages the activation of effector T cells but also increases the number of Tregs, further highlighting the importance of maintaining sufficient glutamine concentration in the immune microenvironment [155]. Therefore, some scholars have proposed the concept of "glutamine hijacking," which suggests that targeted inhibition of glutamine metabolism in tumor cells can alleviate glutamine competition in the tumor microenvironment, release glutamine for immune cell utilization, and promote anti-tumor immune response [134]. These research results provide compelling evidence supporting the view that glutamine competition caused by tumors weakens the immune cell-mediated anti-tumor immunity.

### Tumor-induced glutamine competition via mTOR pathway to regulate immune function

The scarcity of nutrients represents a selective stress that exerts a profound impact on the evolutionary dynamics of an array of cellular processes [156]. Acting as a crucial sensor for assessing the nutritional status of eukaryotic cells, mTOR exhibits activation in response to a surplus of amino acid availability, thereby regulating an assortment of synthetic metabolic pathways essential for sustaining cellular growth [157]. As previously mentioned, immune cells typically lack mechanisms to adapt to nutrient competition, which is a major mechanism regulating anti-tumor immunity. Limited glutamine resources in the microenvironment can inhibit mTOR activity and subsequently affect immune cell function. Figure 4B demonstrates tumor-induced glutamine competition via the mTOR pathway to regulate the function of immune cells. In T cells, inhibition of mTOR activity may lead to compromised activation of c-MYC signaling pathway, causing metabolic stress and affecting T cell proliferation [158]. Additionally, studies have found that mTOR inhibition promotes T cell differentiation towards immunosuppressive Tregs [159]. Another study showed that mTOR inhibition promoted the differentiation of naive CD4 + T cells into Foxp3 + Treg cells with suppressive function in vivo [160]. Conversely, mTOR signaling activation supports the differentiation of naive cells towards Th1 cells [160]. Moreover, in B cells, glutamine deprivation-induced inhibition of mTOR activation can promote GSK3 inhibition and suppress IL-10 secretion [161]. Under low-glutamine conditions, mTOR signaling is significantly suppressed in NK cells, impacting c-MYC expression in IL-2/IL-12-stimulated NK cells and consequently impairing cell growth and NK cell responses [162]. In summary, glutamine deficiency induced by tumor-induced competition highlights the complex role of mTOR in regulating immune cell function.

### Tumor-induced glutamine competition via the PD-1/PD-ligand 1 (PD-L1) axis to regulate immune function

Blockade of the PD-1/PD-L1 axis has brought about a paradigm shift in cancer immunotherapy, demonstrating significant clinical efficacy across a wide range of cancer types [163]. PD-1 is expressed on T cells, while its ligand is expressed on the surface of cancer cells and other cells. This interaction leads to T cell exhaustion and serves as a protective mechanism against T cell-mediated damage to target tissues [164]. The expression of PD-1 and PDL-1 in the tumor immune microenvironment can be influenced by various metabolic factors, including glutamine metabolism [165]. Figure 4C demonstrates that tumor-induced glutamine competition modulates immune function via the PD-1/ PD-L1 axis. When glutamine is restricted, PD-L1 expression is upregulated in tumor cells, which is intriguingly reversible upon glutamine restoration, returning to normal levels [166]. Notably, the deficiency of glutamine can modulate the expression of PD-L1 through multiple pathways [167]. Ma et al. demonstrated that glutamine deprivation activates the EGFR signaling pathway via ERK1/2 and c-Jun activation in renal cancer cells [168]. Treatment with EGFR inhibitor also induced PD-L1 expression and ERK1/2 phosphorylation in renal cancer cells. Importantly, inhibition of EGFR, ERK, and c-Jun phosphorylation suppressed PD-L1 expression induced by glutamine deprivation, suggesting that glutamine deprivation upregulates PD-L1 expression on cancer cells through the EGFR/ERK/c-Jun pathway [168]. In bladder cancer, PD-L1 expression was observed to be upregulated via the EGFR/MEK/ERK/c-Jun pathway under conditions of glutamine deficiency, impairing T cell function [169]. In lung and colon tumors, the inhibition of glutamine utilization led to an increase in PD-L1 expression by reducing GSH levels [170]. This reduction in GSH levels resulted in the inhibition of sarcoplasmic reticulum Ca2 + -ATPase (SERCA) activation, leading to elevated cytosolic Ca^2+^ levels and CaMKII phosphorylation. Subsequently, these changes further activated the downstream NF-kB signaling pathway, ultimately promoting the expression of PD-L1 [170].

Building upon these findings, Zhao et al. developed a photosensitizer-based immunostimulant called BVC, which comprises chlorin e6, an ASCT2 inhibitor (V9302), and a PD-1/PD-L1 blockade agent (BMS-1, 171]. They found that BVC not only enhances the immune recognition between CD8 + T cells and FAS-overexpressing tumor cells but also reduces tumor cell immune escape through PD-1/PD-L1 blockade, resulting in significant benefits for the eradication of metastatic tumors [171]. Recently, researchers have developed nano-bombs for cancer treatment that effectively decrease glutamine levels in cancer cells. This reduction in glutamine subsequently lowers GSH levels and induces endoplasmic reticulum stress, ultimately leading to the upregulation of PD-L1 expression [172]. The intricate interplay between glutamine metabolism and PD-L1 expression in the tumor microenvironment has unveiled novel pathways for therapeutic intervention.

### Tumor-induced glutamine competition promotes glutamate secretion to modulate immune responses

Cancer cells strengthen their resistance to tumor-infiltrating lymphocytes (TILs) partly through the metabolites released into the TME, contributing to its hostility. Multiple studies have indicated that the overexpression of GLS observed in many cancers may not only decrease glutamine levels but also elevate intratumoral glutamate levels [173, 174]. Xiong et al. analyzed the significantly elevated glutameta concentration in tumor tissues of tumor-bearing mice, which was approximately 2–7 times higher than that in adjacent tissues [175]. The breakdown of glutamine into glutamate can have significant effects on immune cells. Figure 4D provides a summary of the impact of tumor cell-secreted glutamine on different immune cells within the TME. Studies have revealed that glutamate can trigger the expression of immunosuppressive genes, including STAT3, ARF4, and RAB10, in neutrophils, which promotes their immunosuppressive phenotype [175]. Importantly, high levels of glutamate in the TME have been found to induce neutrophil transformation into an immunosuppressive state [175]. In MDSCs, the activation of mGluR2/3 reduces the immunosuppressive properties of MDSCs on T cells and diminishes the effects of MDSCs in cancer cells [176, 177]. In T lymphocytes, external application of glutamate has been found to inhibit T cell activation and IL-6 production through the stimulation of metabotropic glutamate receptor 5 [176, 178]. This effect is thought to be mediated by the activation of adenylyl cyclase, leading to the inhibition of multiple signal transduction pathways such as ERK, JNK, and NFκB. Additionally, high levels of glutamate can affect ROS metabolism in T cells by disrupting the function of the Xc^- transporter, which is essential for cysteine uptake and GSH synthesis [179]. In macrophages, the activation of mGluR5 elevates the level of the nuclear receptor peroxisome proliferator-activated receptor (PPAR) g and promotes a shift from M1 to M2 phenotype, leading to reduced IFN-γ production [180]. Tumor-induced competition for glutamine exerts a formidable impact on immune cells, as cancer cells' secretion of glutamate not only disrupts T cell activation and promotes an immunosuppressive phenotype in neutrophils and MDSCs but also facilitates a shift towards an M2 macrophage phenotype. This intricate interplay underscores the pivotal role of glutamate in modulating immune responses within the tumor microenvironment. Targeting glutamate metabolism holds immense potential for enhancing anti-tumor immunity, presenting novel avenues for therapeutic intervention. The detrimental effects of tumor-induced glutamine competition on immune cells cannot be overlooked, and these complex interactions further contribute to providing new paths for therapeutic intervention.

## The role of glutamine metabolism inhibition in modulating immune response

**Fig. 5:**
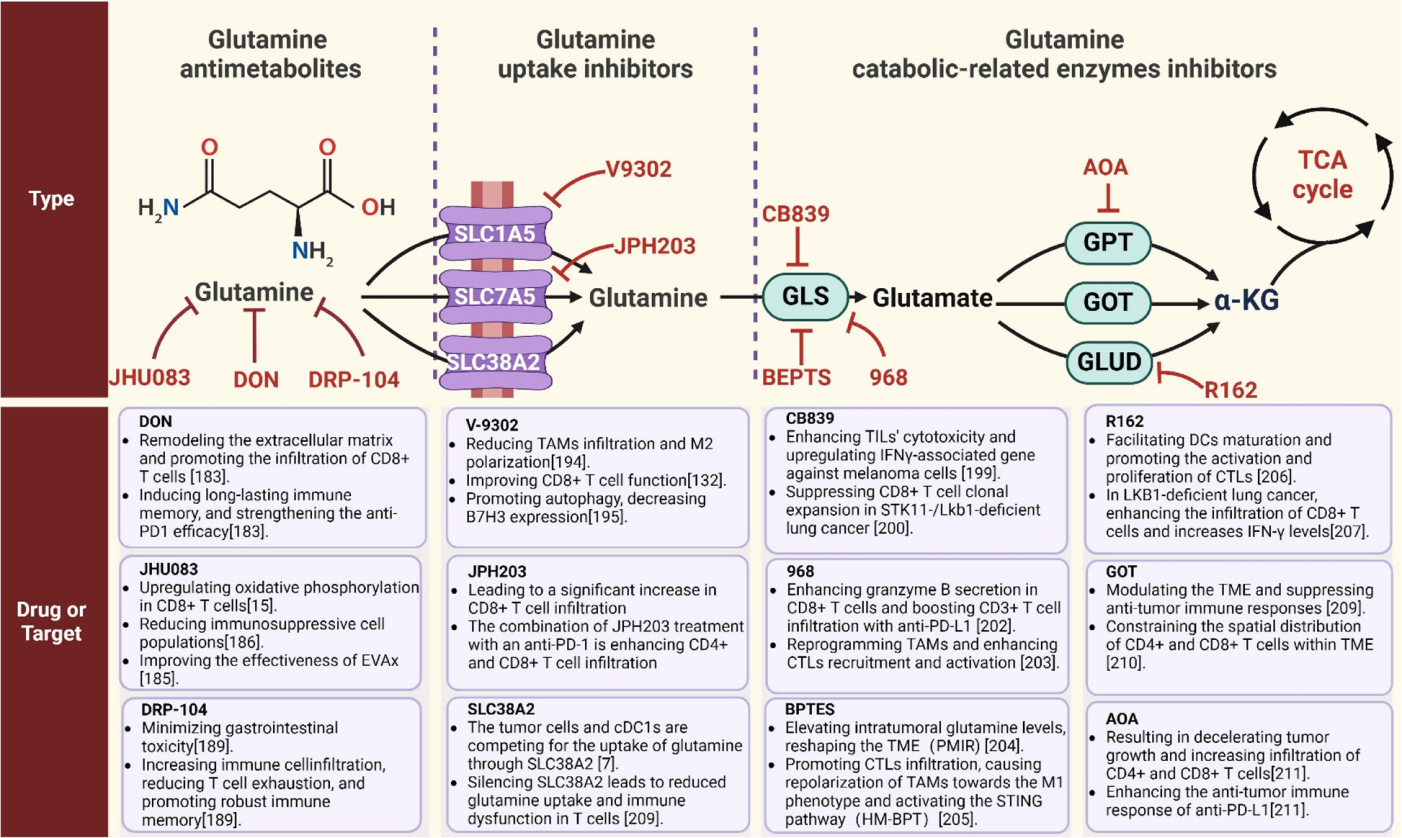
The impact of targeting glutamine metabolism on the tumor immune microenvironment via three distinct avenues: glutamine antimetabolites, inhibitors of glutamine catabolic-related enzymes, and blockers of glutamine uptake. Red solid T arrows direct inhibitory interactions. Created with BioRender.com. GLS, glutaminase; GPT, glutamate pyruvate transaminase; GOT, glutamic-oxaloacetic transaminase; GLUD, glutamate dehydrogenase; CTLs, cytotoxic T lymphocytes; TCA, tricarboxylic acid; TME, tumor microenvironment; AOA: amino-oxyacetic acid

As our comprehension of the intricate nexus between metabolism and immune response progresses, the increasingly discernible efficacy of glutamine metabolism inhibitors as immunomodulatory agents emerges. By selectively targeting pivotal enzymes and pathways implicated in glutamine metabolism, these inhibitors present distinctive opportunities for finely regulating immune activation and suppression. The current repertoire of drugs aimed at glutamine metabolism can be broadly categorized into three classes, specifically glutamine antimetabolites, glutamine catabolic-related enzymes inhibitors, and glutamine uptake inhibitors [181]. Figure 5 presents a comprehensive overview of the impact of inhibiting glutamine metabolism via different pathways on the TME. Further elucidation of the role of glutamine metabolism inhibitors in modulating immune response in cancer paves innovative therapeutic approaches that hold the potential to revolutionize the realm of immunology.

### Glutamine antimetabolites

Glutamine metabolism, since the 1950s, owing to its remarkable antitumor properties [182]. As the critical role of glutamine in TME continues to be unveiled, recent investigations have shifted their attention towards unraveling the intricate involvement of 6-diazo-5-oxo-L-norleucine (DON) in modulating tumor immunity [183]. In pancreatic cancer, DON treatment reduces the levels of hyaluronic acid and collagen, resulting in extensive remodeling of the extracellular matrix (ECM) surrounding the tumor [183]. This remodeling alters the mechanical properties of the ECM, enhancing the infiltration of CD8 + T cells and facilitating the promotion of anti-tumor immune response [183]. Furthermore, DON treatment sensitizes pancreatic cancer to anti-PD-1 therapy, further emphasizing its therapeutic significance [183]. Emerging research on DON and other innovative drugs has unveiled their remarkable potential in harnessing the immune system's power against tumors [184]. In a groundbreaking investigation, Frejlachova et al. discovered that by synergizing DON with an intratumoral immunotherapy approach utilizing mannan-BAM, TLR ligands, and anti-CD40 antibody, they achieved a staggering 50% complete eradication of advanced Panc02 subcutaneous tumors in treated animals [184]. Notably, this therapeutic combination also induced long-lasting immune memory [184]. These findings underscore the promising role of DON in empowering anti-tumor immunity and open new avenues for future therapeutic interventions.

JHU083, an orally active precursor to a glutamate antagonist, selectively activates within the tumor microenvironment, leading to glutamine metabolism inhibition [185]. In CD8 + T cells, JHU-083 suppresses glutamine metabolism and glycolysis but upregulates OXPHOS, utilizing extracellular acetate as an alternative fuel to potentiate its anti-tumor effects [15]. Moreover, JHU083 modulates MDSCs within the TME, promoting their polarization into pro-inflammatory TAMs and inhibiting the expression of IDO, thereby boosting the anti-tumor immune response [147]. Importantly, JHU083 treatment reduces immunosuppressive cell populations in murine models, including monocyte and granulocyte-derived suppressive cells, regulatory T cells, and pro-tumor CD4 + Th17 cells [185]. These findings provide compelling evidence of JHU083’s ability to enhance immune cell activity, thereby improving the effectiveness of immunotherapy [185]. Moreover, under glutamine inhibition conditions, Th1 cells display heightened oxidative metabolism and exhibit a highly activated, memory-like phenotype, presenting new avenues for exploration. Excitingly, combining JHU083 treatment with the EGFR peptide vaccine (EVAx) significantly inhibits lung tumor development, highlighting the potential synergy of this combination therapy in promoting tumor-specific immune responses mediated by adaptive T cells [185]. Furthermore, JHU083 has been reported to enhance the proliferation of CD8 + T cells and improve the efficacy of PD-1 blockade in hepatocellular carcinoma [151].

DRP-104 (sirpiglenastat) represents a novel prodrug of the broad-spectrum glutamine antagonist DON. As an inactive form, DRP-104 preferentially converts to DON within tumors [186]. Unlike DON, which can induce gastrointestinal toxicity upon administration, DRP-104 undergoes inert metabolic inactivation in gastrointestinal tissues. It has been reported that DRP-104 achieves 11-fold higher exposure of DON in tumor tissues compared to gastrointestinal tissues, highlighting its immense potential and stimulating further investigation into its anti-tumor effects [187]. Metabolomic analysis of tumors treated with DRP-104 reveals extensive alterations, indicative of dysregulation in tumor synthetic metabolism and classical cancer metabolic pathways, including changes in glutamine metabolism and reduction in several immunosuppressive metabolites [186]. Specifically, treatment with DRP-104 results in significant increases in various infiltrating immune cells, such as T cells, NK cells, and NKT cells [186]. Functionally, T cells exhibit enhanced proliferative capacity and reduced exhaustion, while tumor-associated macrophages polarize towards the M1 phenotype [186]. There is a reduction in myeloid-derived suppressor cells and pro-tumor proteins within the TME [186]. Ultimately, as a monotherapy, DRP-104 demonstrates notable anti-tumor activity, with further enhanced therapeutic efficacy when used in combination with checkpoint blockade therapy, leading to improved survival and long-term durable cures [186]. Moreover, researchers have discovered that the effects of DRP-104 are CD8 + T cell-dependent and result in robust immune memory [187]. A recent study has reported that DRP-104 exerts its effects in KEAP1 mutant lung cancer by suppressing glutamine-dependent nucleotide synthesis. Notably, DRP-104 demonstrates the ability to counter T cell exhaustion and enhance the functionality of both CD4 + and CD8 + T cells [188]. The discovery and application of agents like DRP-104 open up new avenues and possibilities for the development of more effective cancer treatment strategies.

### Glutamine uptake inhibitors

In an ideal scenario, tumor cells upregulate specific glutamine transporters, leading to a reduction in glutamine levels within the tumor microenvironment and depriving immune cells of their glutamine demand. This creates a competition for glutamine between tumor cells and immune cells. By inhibiting the ability of tumor cells to uptake glutamine and increasing the levels of glutamine within the tumor microenvironment, a dual effect can be achieved: suppressing tumor cell growth on one hand and enhancing the availability of glutamine for immune cells on the other hand, thereby strengthening the anti-tumor immune function of immune cells. The inhibition of certain AATs has demonstrated a promising anti-tumor immune response. SLC1A5 is a crucial sodium-dependent solute carrier protein that facilitates the import of glutamine and serves as the principal conduit through which cancer cells acquire glutamine [189, 190]. Researchers have discovered a potent and selective glutamine transporter inhibitor, V9302, which specifically targets the SLC1A5 and effectively inhibits glutamine uptake [191].SLC1A5 has been reported to be involved in the modulation of the immune microenvironment in multiple types of tumors. In glioma, ASCT2 has been linked to immune response, and its knockdown effectively reduces tumor-associated macrophage infiltration as well as M2 polarization in glioma [192]. In TNBC, V9302 improved CD8 + T cell effector function by driving GSH synthesis through selective inhibition of glutamine uptake by TNBC cells [134]. Furthermore, V9302 exerts its impact on the immune microenvironment through another pathway, promoting autophagy to regulate the accumulation of ROS [193]. This leads to a decrease in B7H3 expression and an enhancement in the production of granzyme B in CD8 + T cells. In a syngeneic mouse model, the combination therapy of V9302 and anti-PD-1 demonstrates the ability to enhance antitumor immunity [193]. Recently, a groundbreaking therapeutic strategy utilizing molybdenum disulfide (MoS2) as a carrier for the delivery of anti-PD-L1 antibody and V9302, a competitive transmembrane antagonist targeting the amino acid transporter ASCT2, has demonstrated remarkable potential in enhancing anti-tumor immune responses within TNBC cells [194]. Specifically, this treatment facilitates the migration of CD8 + T cells from the tumor periphery into the tumor core, effectively impeding the growth of TNBC 4T1 tumors [194]. Notably, following treatment with MoS2-aPD-L1-V9302, there is a substantial increase in glutamine levels within the tumor interstitial fluid, resulting in a significant augmentation of activated T cells [194]. SLC7A5 functions as an antiporter, facilitating the exchange of intracellular amino acids, specifically glutamine, with a 1:1 stoichiometry across the cell membrane [195]. Recent findings have shown that reducing the expression of SLC7A5 in TNBC can lead to a remarkable increase in the infiltration of CD8 + T cells [196]. Building upon this discovery, a promising therapeutic approach has emerged, involving the combination of JPH203 treatment, which effectively blocks SLC7A5, with an anti-PD-1 antibody. This powerful combination demonstrates synergistic effects, bolstering CD4 + T and CD8 + T cells infiltration and significantly impeding the progression of tumors. These breakthroughs not only deepen our understanding of the intricate mechanisms at play in TNBC but also offer a potential avenue for enhancing immunotherapy efficacy by strategically targeting SLC7A5 [196]. These findings highlight the therapeutic potential of targeting glutamine transporters and its impact on the immune microenvironment in cancer treatment.

However, it is worth noting that certain known glutamine transporters not only function in tumor cells but also play an important role in immune cells. Consequently, the current inhibitors may potentially impact the glutamine transport in immune cells, thereby attenuating the anti-tumor immune response. SLC38A2, a secondary active transporter energized by the Na + electrochemical gradient, facilitates the transport of neutral amino acids, particularly in glutamine transportation [197]. A recent study explored the mechanisms underlying the functional impairment and metabolic reprogramming of T lymphocytes in multiple myeloma and found silencing SLC38A2 resulted in decreased glutamine uptake and immune dysfunction of T cells [198]. Another study explored the expression patterns of SLC38A2 in both tumor and immune cells, revealing significantly higher expression levels in tumor cells and dendritic cells compared to CD8 + T cells and other immune cells [7]. Both tumor cells and cDC1s engage in competitive uptake of glutamine through the SLC38A2 transporter protein, and the lack of SLC38A2 compromises the ability of DCs to promote T cell priming [7]. In summary, developing inhibitors targeting nutrient transporters has emerged as a promising strategy to effectively weaken cancer cell metabolism and block tumor growth. However, these inhibitors may also impact nutrient transporters on immune cells, leading to immunosuppression. This dual effect underscores the importance of targeting specific transporters on cancer cells. Thus, developing inhibitors specifically targeting nutrient transporters on cancer cells would be the most ideal choice. One exceptional example is that the application of pharmacological intervention with the SLC1A5 inhibitor V9302 demonstrated remarkable selectivity in blocking glutamine uptake specifically in TNBC cells, while leaving CD8 + T cells unaffected [134]. Intriguingly, CD8 + T cells exhibit an adaptive response by upregulating an alternative glutamine transporter, SLC6A14, thereby sustaining both glutamine uptake and immune effector function within V9302-treated tumors [134]. This finding not only expands our understanding of the complexity of targeting glutamine transporters, but also provides new avenues and possibilities for customizing more precise treatment strategies that can be tailored to individual patients.

### Glutamine catabolic-related enzymes inhibitors

The critical role of GLS as a key enzyme involved in glutamine metabolism has emerged as a promising approach for cancer therapy. GLS is an enzyme that converts glutamine to glutamate, and small molecule inhibitors targeting GLS include CB-839, 968 and BPTES. The role of CB839 in the tumor immune microenvironment has been extensively evaluated in preclinical experiments, revealing its distinct effects within the TME. For instance, CB-839 enhances the cytotoxicity of autologous TILs against melanoma cells derived from patients [199]. Furthermore, when combined with anti-PD1 or anti-CTLA4 antibodies, CB-839 promotes the infiltration of effector T cells into tumors and upregulates IFNγ-associated gene expression, thereby enhancing the antitumor activity of these checkpoint inhibitors [199]. In STK11-/Lkb1-deficient lung cancer, CB839 demonstrates a notable suppression of CD8 + T cells clonal expansion and activation [200]. Recently, researchers have successfully engineered a dual-mechanism-based nutrient partitioning nanoregulator (DMNPN) that downregulates glutaminase expression and enhances nutrient availability for T cells, thereby bolstering their antitumor immunity [201]. Compound 968, a promising agent currently undergoing preclinical evaluation, exhibits potent inhibition of ovarian cancer cell proliferation while enhancing granzyme B secretion in CD8 + T cells [202]. Combination therapy with anti-PD-L1 and compound 968 results in increased secretion of granzyme B, CXCL10, and CXCL11 by CD4 + and CD8 + T cells, thereby bolstering CD3 + T cell infiltration into tumor microenvironments [202]. In recent investigations, researchers have developed an innovative combination of compound 968 and photosensitizers. This groundbreaking approach induces immunogenic cell death in tumor cells, leading to the generation of novel antigens that facilitate dendritic cell maturation [203]. Consequently, this process orchestrates the recruitment and activation of cytotoxic T lymphocytes (CTLs) [203]. Moreover, the intervention of C9SN remodels the TME by targeting glutamine metabolism, polarizing M2 TAMs towards an M1 phenotype. This reprogramming further enhances the recruitment and activation of CTLs [203]. The past years have seen significant progress in the development of GLS inhibitor, BPTES, as a promising drug candidate by researchers. Researchers present a metabolically and immunologically targeted agent, termed PD-L1-targeted metabolism and immune regulator (PMIR), constructed using a PD-L1-targeting peptide modified glutaminase inhibitor (BPTES) loaded into zeolitic imidazolate framework [204]. At the tumor site, PMIR effectively inhibits glutamine metabolism in tumor cells, leading to an elevation in intratumoral glutamine levels and improving immune cell functionality, ultimately reshaping the immunosuppressive TME and evoking robust immune responses [204]. In addition, researchers have developed a multifunctional biomimetic nanoplatform (referred to as HM-BPT), which utilizes pH-sensitive tumor-targeting hybrid membrane-coated manganese oxide nanoparticles for the delivery of the glutaminase inhibitor BPTES. Treatment with HM-BPT in a 4T1 xenograft model resulted in significant tumor suppression, infiltration of CTLs, as well as M1 phenotype repolarization and activation of the stimulator of IFN genes (STING) pathway in macrophages [205]. These studies highlight the potential of GLS inhibitors in cancer therapy, providing insights into the modulation of the immune microenvironment and the development of innovative treatment strategies.

GLUD1, a critical enzyme involved in glutamine metabolism within mitochondria, has recently emerged as a key player in shaping the tumor immune microenvironment. The innovative therapeutic approach employing MLipRIR-NPs combines R162, a glutamate dehydrogenase inhibitor, and the hydrophobic photosensitizer IR780 [206]. This strategy effectively disrupts the glutamine metabolism pathway within mitochondria, leading to cancer cell immunogenic death [206]. Importantly, GLUD1 inhibition through MLipRIR-NPs treatment has been shown to facilitate dendritic cell maturation, a process essential for antigen presentation and immune cell activation [206]. The activated dendritic cells promote the activation and proliferation of CTLs within tumors [206]. This dual mechanism not only induces tumor cell death but also enhances the antitumor immune response [206]. Furthermore, combining GLUD1 inhibition with an anti-PD-L1 antibody amplifies the therapeutic effects. This combination therapy results in increased activation of CD8 + T cells and CD86 + dendritic cells in the spleen, leading to elevated levels of pro-inflammatory cytokines [206]. Moreover, recent studies have revealed the association between GLUD1 and ribosomal S6 kinase 2 (RSK2) in LKB1-deficient lung cancer patients [207]. Inhibiting GLUD1/RSK2 has been shown to enhance the infiltration of CD8 + T cells and increase IFN-γ levels within tumors, indicating the critical role of GLUD1 in shaping the immune microenvironment in specific cancer subtypes [207]. These findings highlight the importance of GLUD1 in shaping the immune microenvironment and pave the way for the development of tailored immunotherapeutic approaches.

GOT exists in two distinct forms: cytoplasmic GOT1 and mitochondrial GOT2 [197]. The transaminase activity of GOT2 facilitates the conversion of glutamate and OAA into α-KG and aspartate, while GOT1 catalyzes the reverse reaction [198]. GOT2 has been identified as playing a pivotal role in pancreatic cancer, modulating the immune microenvironment and suppressing anti-tumor immune responses. Beyond its conventional malate-aspartate shuttle function, GOT2 also enhances the transcriptional activity of the PPARδ through direct fatty acid binding. While GOT2 is not essential for in vivo proliferation of cancer cells, its interaction with PPARδ constrains the spatial distribution of CD4 + and CD8 + T cells within the tumor microenvironment. This novel insight offers a fresh perspective on the mechanisms of tumor immune evasion and provides critical clues for the development of therapeutic strategies targeting the immune microenvironment [208]. GPT2, another key enzyme facilitating the transamination between alanine and α-ketoglutarate, has recently been reported to be involved in the formation of the tumor immune microenvironment [209]. Aminooxyacetic acid hemihydrochloride (AOA), a notable GPT2 inhibitor, exhibits promising effects [210]. In this groundbreaking study, the authors have successfully demonstrated that the utilization of AOA results in a remarkable reduction in tumor growth rate and an impressive enhancement in the infiltration of CD4 + and CD8 + T cells [209]. Notably, when combined with anti-PD-L1 treatment, a synergistic effect is observed, leading to a substantial potentiation of the anti-tumor immune response [209]. These findings shed new light on the therapeutic potential of AOA as a valuable intervention for combating cancer [209]. These compelling findings underscore the importance of glutamine metabolic enzymes as gatekeepers of immune surveillance and anti-tumor immunity. Future research endeavors should continue to elucidate these discoveries, delving deeper into the molecular intricacies that the interplay between glutamine metabolism and the immune microenvironment.

### Novel approach to inhibit glutamine metabolism

In recent years, there has been a growing interest in developing novel approaches to manipulate glutamine metabolism and modify the immune microenvironment to enhance the cytotoxicity of immune cells against tumors. A recent study describes a pioneering strategy involving a newly developed polymer containing hydrophobic ferrocene units and thioketal bonds in the main chain. This polymer successfully delivered a prodrug of oxaliplatin and artesunate, referred to as Artoxplatin, to cancer cells [172]. Metabolomics analysis revealed a decrease in glutamine levels in the cancer cells treated with this novel polymer, which led to an upregulation of PD-L1 [172]. Importantly, combining the use of this polymer, named nanobombig, with anti-PD-L1 therapy demonstrated enhanced tumor inhibitory effects [172]. Furthermore, researchers discovered that osmundacetone (OSC), a bioactive phenolic compound derived from Phellinus igniarius, exhibited regulatory effects on the immune microenvironment, as confirmed by downregulation of GLUD1 expression upon OSC treatment [211]. Additionally, concentrations of related metabolites α-KG and NADH were reduced in response to OSC treatment [211]. However, further investigation is needed to fully elucidate the immunomodulatory role of OSC in the microenvironment.

#### Recent advances in clinical trials of glutamine metabolism inhibitors

In the landscape of oncological research, the inhibition of glutamine metabolism has emerged from preclinical studies as a compelling avenue for therapeutic intervention, sparking fervent interest in its clinical application. This surge of enthusiasm is bolstered by the ongoing scrutiny of 24 clinical trials cataloged on ClinicalTrials.gov as of November 26, 2023 (Table 2), which are dedicated to probing the pharmacological properties of glutaminase inhibitors and delving into their immunogenic characteristics.

**Table 2:** Characteristics of ClinicalTrials.gov registered glutamine metabolic interventions for cancer therapy

**Therapeutic agents**	**Cancer type**	**NCT member**	**Phase**	**Combination therapy**	**Status**
CB-839	Leukemia	NCT02071927	I	None	Completed
CB-839	Hematological tumors	NCT02071888	I	None	Completed
CB-839	Solid tumors	NCT02071862	I	None	Completed
CB-839	Solid tumors and fluoropyrimidine resistant PIK3CA mutant colorectal cancer	NCT02861300	I; II	None	Not yet recruiting
CB-839	Advanced stage non-small cell lung cancer	NCT04250545	I	Sapanisertib	Suspended (Drug supply issues)
CB-839	Solid tumors	NCT03875313	I; II	None	Completed (Slow enrollment)
CB-839	Recurrent or refractory Multiple Myeloma	NCT03798678	I	Carfilzomib	Active
CB-839	Metastatic and refractory RAS wildtype colorectal cancer	NCT03263429	I; II	Panitumumab; Irinotecan	Active
CB-839	Platinum resistant BRCA -wild-type ovarian cancer	NCT03944902	I	Niraparib	Terminated (Participant off study)
CB-839	Solid tumors	NCT03965845	I; II	Palbociclib	Completed
CB-839	EGFR-mutated stage IV non-small cell lung cancer	NCT03831932	I; II	Osimertinib	Recruiting
CB-839	Advanced triple negative breast cancer	NCT03057600	II	Paclitaxel	Completed
CB-839	IDH-mutated diffuse astrocytoma or anaplastic astrocytoma	NCT03528642	I	Radiation Therapy	Active
CB-839	Renal cell carcinoma	NCT03163667	II	Everolimus	Completed
CB-839	Prostate cancer	NCT04824937	II	Talazoparib	Unknown
CB-839	Metastatic renal cell carcinoma	NCT03428217	II	Cabozantinib	Completed
CB-839	Melanoma; Clear cell renal cell carcinoma; Non-small cell lung cancer	NCT02771626	I; II	Nivolumab	Terminated (Lack of efficacy)
CB-839	Advanced solid tumor	NCT03872427	II	No	Active
CB-839	Advanced cervical cancer	NCT05521997	II	Chemoradiation	Not yet recruiting
CB-839	KEAP1 or NRF2-mutated, non-squamous NSCLC	NCT04265534	II	Pembrolizumab	Terminated (Lack of efficacy)
IPN60090	Advanced solid tumors	NCT03894540	II	Pemobrolizumab	Terminated
IPN60090	Advanced solid tumors	NCT05039801	I	Bevacizumab; Paclitaxel	Recruiting
DRP-104	Advanced solid tumors	NCT04471415	I; II	Atezolizumab	Terminated
DRP-104	Fibrolamellar hepatocellular carcinoma	NCT06027086	I; II	Durvalumab	Not yet recruiting

Among these inhibitors, CB-839 has risen as a beacon of promise, venturing into extensive Phase I/II clinical trials [212, 213]. Multiple clinical trial findings have illuminated the favorable clinical activity of CB-839 alongside minimal adverse reactions [212–216]. Furthermore, investigations into the combination of CB-839 with immunotherapy have yielded encouraging outcomes, exemplified by its synergistic effect with nivolumab in patients diagnosed with melanoma, clear cell renal cell carcinoma, and NSCLC [217]. Conversely, while certain trials exploring CB-839 alone or in combination with other therapies have exhibited less auspicious results, the potential value of CB-839 as a complementary component to existing treatment paradigms persists [218]. Meanwhile, IPN60090, also known as IACS-6274, has emerged as another promising GLS1 inhibitor, boasting favorable physicochemical properties and pharmacokinetic characteristics, coupled with a remarkable affinity for the target molecule [219, 220]. Ongoing clinical trials are actively unveiling insights into the prospective applications of IPN60090 in cancer therapy, shedding light on its potential therapeutic significance (NCT03894540, NCT05039801). In a parallel vein, the novel prodrug of the broad-spectrum glutamine antagonist DON is DRP-104, alias sirpiglenastat, stands out as a beacon of promise in the realm of anti-tumor agents [187]. Its distinctive inactive-to-active conversion within the tumor microenvironment augments metabolic targeting and selectivity, which underscores its potential as a groundbreaking therapeutic strategy [186, 187]. Currently, two clinical trials are underway to assess its safety, tolerability, and efficacy (NCT04471415, NCT06027086). The inhibition of glutamine metabolism has emerged as a promising avenue for cancer therapy, with CB-839, IPN60090, and DRP-104 representing exciting new drugs that hold significant potential. While clinical trials have shown promising results, it is important to continue exploring these compounds and their precise mechanisms of action, as well as identifying the patient populations who may benefit the most from these therapies.

#### Unresolved questions and future directions

Currently, despite promising prospects in preclinical models, targeting glutamine metabolism faces challenges in clinical trials due to inherent heterogeneity in glutamine metabolism within tumor cells and immune cells, as well as its complex impact on immune responses. As is well known, TME is a dynamic ecosystem with spatiotemporal variations [221]. Investigating how glutamine metabolism adapts to the constantly changing TME clues, such as hypoxia, acidosis, and nutrient deprivation, provides crucial insights into understanding immune cell function and resistance mechanisms [222, 223]. Recent studies have reported the various strategies used to inhibit glutamine metabolism have discrete effects on CD8 + T cells, emphasizing that targeting the same pathway with different modes may result in conflicting metabolic and functional consequences, further highlighting the importance of understanding how glutamine precisely affects the metabolic pathways and functional dynamics of different immune cell subpopulations in TME [224]. Organoid and microfluidic platforms provide new opportunities for studying TME in a controlled manner. Using these advanced models, researchers are expected to dissect the complex interactions between immune cells and tumor cells, ultimately revealing the exact role of glutamine metabolism in shaping the immune landscape. Furthermore, considering the widespread importance of glutamine in various cellular metabolic processes, potential therapeutic approaches targeting glutamine metabolism may have unexpected consequences on immune cells. Early clinical trials of glutamine antagonist metabolites have revealed unacceptable systemic toxicity, highlighting the need for more selective approaches to disrupt glutamine metabolism in cancer patients [225]. While there are promising therapeutic targets within glutamine metabolism, selectively inhibiting glutamine metabolism in cancer cells while preserving normal tissues remains a major challenge. Overcoming this obstacle requires innovative strategies that exploit the unique metabolic vulnerabilities of tumors to ensure optimal efficacy and minimal off-target effects. Furthermore, the identification of additional metabolic regulators and signaling pathways that regulate glutamine metabolism in cancer and immune cells presents a feasible solution. Further fundamental research is needed to unravel the mechanisms underlying glutamine regulation, which could unlock new therapeutic targets and provide deeper insights into the complex interactions within the tumor microenvironment. In the near future, the development of specific inhibitors targeting glutamine metabolism in cancer cells will be an ideal choice. Moreover, exploring the interplay between glutamine metabolism and other key metabolic pathways, such as glucose metabolism, lipid metabolism, and amino acid metabolism, remains an actively researched area. Deciphering the intricate connections and dependencies among these metabolic pathways will help discover novel metabolic targets and pathways, paving the way for synergistic therapeutic opportunities and innovative treatment modalities. Integrating multi-omics data, including genomics, transcriptomics, proteomics, and metabolomics, with advanced computational approaches will provide a comprehensive framework for unraveling the complex networks and interactions involving glutamine metabolism. Such integrative data analysis methods can reveal new therapeutic targets and guide the development of innovative treatment strategies. Although there are challenges and unanswered questions in the field of targeting glutamine metabolism, ongoing research provides promising avenues for future exploration. Continued efforts in this field hold great potential for advancing precision medicine and improving patient outcomes.

## Conclusion

Glutamine metabolism is a crucial battleground in the fight against cancer as cancer cells rely on glutamine as an essential energy source, while immune cells demand it during their activation and proliferation in immune responses. This metabolic competition for limited resources is a complex and fluctuating struggle between opposing factions that has significant implications for tumor control and immune surveillance. Recent advances have shed light on the critical role of dysregulated glutamine metabolism in cancer development and immune responses. However, a comprehensive understanding of the intricate molecular mechanisms involved is still lacking. Targeting key components of glutamine metabolism, such as transporters and enzymes, presents an exciting opportunity to enhance anti-tumor immunity by tipping the balance in favor of immune cells. Nevertheless, the translation of these findings into effective clinical interventions is still in its early stages. Further investigation of the intricate molecular mechanisms underlying this ongoing battle is essential for the development of innovative therapeutic approaches that enhance anti-tumor immunity and lead to improved patient outcomes. The ongoing exploration of these multifaceted molecular pathways not only deepens our understanding of this glutamine tug-of-war but also paves the way for pioneering interventions that have the power to revolutionize the landscape of cancer treatment.

## Data Availability

Not applicable.

## Abbreviations

AATs: Amino acid transporters
AOA: Aminooxyacetic acid hemihydrochloride
ASCT2: Alanine-serine-cysteine transporter 2
ATB0, +: Amino acid transporter B0, +
ATP: Adenosine triphosphate
CPSI: Carbamoyl phosphate synthetase I
CPSII: Carbamoyl phosphate synthetase II
CRC: Colorectal cancer
CTLs: Cytotoxic T lymphocytes
CTPS: Cytidine triphosphate synthetase
DMNPN: Dual-mechanism-based nutrient partitioning nanoregulator
ER +: Estrogen receptor positive
FADH2: Flavin adenine dinucleotide
FGAR: Formylglycinamide ribonucleotide
GLS: Glutaminase
GLUD: Glutamate dehydrogenase
GOT: Glutamate-oxaloacetate transamininase
GPT: Glutamate pyruvate transaminase
GSH: Glutathione
HNSCC: Head and neck squamous cell carcinoma
IFN: Interferons
Ig: Immunoglobulin
IL: Interleukin
KG: Ketoglutarate
LAT1: L-amino acid transporter 1
MDSCs: Myeloid-derived suppressive cells
mTOR: Mechanistic target of rapamycin
NAD: Nicotinamide adenine dinucleotide
NADH: Reduced nicotinamide adenine dinucleotide
NF-KB: Nuclear factor-kappa B
NK: Natural killer
NSCLC: Non-small cell lung cancer
OSC: Osmundacetone
OXPHOS: Oxidative phosphorylation
PD-1: Programmed cell death- 1
PD-L1: Programmed cell death- ligand 1
PMIR: PD-L1-targeted metabolism and immune regulator
PPAR: Peroxisome proliferator-activated receptor
PPAT: Phosphoribosyl pyrophosphate amidotransferase
PPP: Pentose phosphate pathway
PRA: Phosphoribosyl-β-amine
PRPP: Phosphoribosyl-α-pyrophosphate
RACK1: Receptor for activated C kinase 1
ROS: Reactive oxygen species
RSK2: Ribosomal S6 kinase 2
SERCA: Sarcoplasmic reticulum Ca2 + -ATPase
SNAT1: Sodium-coupled neutral amino acid transporter 1
TAMs: Tumor-associated macrophages
TANs: Tumor-associated neutrophils
TCA: Tricarboxylic acid
TGF: Transforming growth factor
TILs: Tumor-infiltrating lymphocytes
TME: Tumor microenvironment
TNBC: Triple-negative breast cancer
TNF: Tumour necrosis factor
Tregs: Regulatory T cells
UTP: Uridine triphosphate
OSC: Osmundacetone

## References

[1] Stine ZE, Schug ZT, Salvino JM, Dang CV (2022). Targeting cancer metabolism in the era of precision oncology. Nat Rev Drug Discov.

[2] Koppenol WH, Bounds PL, Dang CV (2011). Otto Warburg’s contributions to current concepts of cancer metabolism. Nat Rev Cancer.

[3] Pantel AR, Ackerman D, Lee SC, Mankoff DA, Gade TP (2018). Imaging cancer metabolism: underlying biology and emerging strategies. J Nucl Med.

[4] Reinfeld BI, Madden MZ, Wolf MM, Chytil A, Bader JE, Patterson AR (2021). Cell-programmed nutrient partitioning in the tumour microenvironment. Nature.

[5] Fu Q, Xu L, Wang Y, Jiang Q, Liu Z, Zhang J (2019). Tumor-associated macrophage-derived interleukin-23 interlinks kidney cancer glutamine addiction with immune evasion. Eur Urol.

[6] Kerk SA, Lin L, Myers AL, Sutton DJ, Andren A, Sajjakulnukit P (2022). Metabolic requirement for GOT2 in pancreatic cancer depends on environmental context. Elife..

[7] Guo C, You Z, Shi H, Sun Y, Du X, Palacios G (2023). SLC38A2 and glutamine signalling in cDC1s dictate anti-tumour immunity. Nature.

[8] Pavlova NN, Zhu J, Thompson CB (2022). The hallmarks of cancer metabolism: still emerging. Cell Metab.

[9] Krebs HA (1935). Metabolism of amino-acids: the synthesis of glutamine from glutamic acid and ammonia, and the enzymic hydrolysis of glutamine in animal tissues. Biochem J.

[10] Kovacevic Z, Morris HP (1972). The role of glutamine in the oxidative metabolism of malignant cells. Cancer Res.

[11] Reitzer LJ, Wice BM, Kennell D (1979). Evidence that glutamine, not sugar, is the major energy source for cultured HeLa cells. J Biol Chem.

[12] Ardawi MS, Newsholme EA (1983). Glutamine metabolism in lymphocytes of the rat. Biochem J.

[13] Lobo C, Ruiz-Bellido MA, Aledo JC, Marquez J, Nunez De Castro I, Alonso FJ. Inhibition of glutaminase expression by antisense mRNA decreases growth and tumourigenicity of tumour cells. Biochem J. 2000;348 Pt 2(Pt 2):257–61. PMC1221061 10816417

[14] Gross MI, Demo SD, Dennison JB, Chen L, Chernov-Rogan T, Goyal B (2014). Antitumor activity of the glutaminase inhibitor CB-839 in triple-negative breast cancer. Mol Cancer Ther.

[15] Leone RD, Zhao L, Englert JM, Sun IM, Oh MH, Sun IH (2019). Glutamine blockade induces divergent metabolic programs to overcome tumor immune evasion. Science.

[16] Li M, Yang Y, Xiong L, Jiang P, Wang J, Li C (2023). Metabolism, metabolites, and macrophages in cancer. J Hematol Oncol.

[17] Altman BJ, Stine ZE, Dang CV (2016). From Krebs to clinic: glutamine metabolism to cancer therapy. Nat Rev Cancer.

[18] Andrejeva G, Rathmell JC (2017). Similarities and distinctions of cancer and immune metabolism in inflammation and tumors. Cell Metab.

[19] Buck MD, O'Sullivan D, Pearce EL (2015). T cell metabolism drives immunity. J Exp Med.

[20] Kedia-Mehta N, Finlay DK (2019). Competition for nutrients and its role in controlling immune responses. Nat Commun.

[21] Ma G, Zhang Z, Li P, Zhang Z, Zeng M, Liang Z (2022). Reprogramming of glutamine metabolism and its impact on immune response in the tumor microenvironment. Cell Commun Signal.

[22] Pallett LJ, Dimeloe S, Sinclair LV, Byrne AJ, Schurich A (2021). A glutamine 'tug-of-war': targets to manipulate glutamine metabolism for cancer immunotherapy. Immunother Adv..

[23] Zhu L, Zhu X, Wu Y (2022). Effects of glucose metabolism, lipid metabolism, and glutamine metabolism on tumor microenvironment and clinical implications. Biomolecules..

[24] Sharma S, Agnihotri N, Kumar S (2022). Targeting fuel pocket of cancer cell metabolism: a focus on glutaminolysis. Biochem Pharmacol.

[25] Kerr MC, Teasdale RD (2009). Defining macropinocytosis. Traffic.

[26] Rahl PB, Lin CY, Seila AC, Flynn RA, McCuine S, Burge CB (2010). c-Myc regulates transcriptional pause release. Cell.

[27] Wise DR, Thompson CB (2010). Glutamine addiction: a new therapeutic target in cancer. Trends Biochem Sci.

[28] Hensley CT, Wasti AT, DeBerardinis RJ (2013). Glutamine and cancer: cell biology, physiology, and clinical opportunities. J Clin Invest.

[29] Bhutia YD, Babu E, Ramachandran S, Ganapathy V (2015). Amino Acid transporters in cancer and their relevance to “glutamine addiction”: novel targets for the design of a new class of anticancer drugs. Cancer Res.

[30] Scalise M, Pappacoda G, Mazza T, Console L, Pochini L, Indiveri C (2022). Cysteine 467 of the ASCT2 amino acid transporter is a molecular determinant of the antiport mechanism. Int J Mol Sci..

[31] Sastrasinh M, Sastrasinh S (1990). Effect of acute pH change on mitochondrial glutamine transport. Am J Physiol.

[32] van Geldermalsen M, Wang Q, Nagarajah R, Marshall AD, Thoeng A, Gao D (2016). ASCT2/SLC1A5 controls glutamine uptake and tumour growth in triple-negative basal-like breast cancer. Oncogene.

[33] Toda K, Nishikawa G, Iwamoto M, Itatani Y, Takahashi R, Sakai Y (2017). Clinical role of ASCT2 (SLC1A5) in KRAS-mutated colorectal cancer. Int J Mol Sci..

[34] Lin J, Yang T, Peng Z, Xiao H, Jiang N, Zhang L (2018). SLC1A5 silencing inhibits esophageal cancer growth via cell cycle arrest and apoptosis. Cell Physiol Biochem.

[35] Wu J, Li Z, Yang Z, Guo L, Zhang Y, Deng H (2018). A Glutamine-rich carrier efficiently delivers anti-CD47 siRNA driven by a “Glutamine Trap” to inhibit lung cancer cell growth. Mol Pharm.

[36] Guo H, Xu Y, Wang F, Shen Z, Tuo X, Qian H (2018). Clinical associations between ASCT2 and p-mTOR in the pathogenesis and prognosis of epithelial ovarian cancer. Oncol Rep.

[37] Wang Q, Hardie RA, Hoy AJ, van Geldermalsen M, Gao D, Fazli L (2015). Targeting ASCT2-mediated glutamine uptake blocks prostate cancer growth and tumour development. J Pathol.

[38] Liu Y, Yang L, An H, Chang Y, Zhang W, Zhu Y (2015). High expression of Solute Carrier Family 1, member 5 (SLC1A5) is associated with poor prognosis in clear-cell renal cell carcinoma. Sci Rep.

[39] Zhang Z, Liu R, Shuai Y, Huang Y, Jin R, Wang X (2020). ASCT2 (SLC1A5)-dependent glutamine uptake is involved in the progression of head and neck squamous cell carcinoma. Br J Cancer.

[40] Jiang J, Dong W, Zhang W, Wang Q, Wang R, Wang J (2023). LncRNA SLC1A5-AS/MZF1/ASCT2 axis contributes to malignant progression of hepatocellular carcinoma. Discov Med.

[41] Fotiadis D, Kanai Y, Palacin M (2013). The SLC3 and SLC7 families of amino acid transporters. Mol Aspects Med.

[42] Fuchs BC, Bode BP (2005). Amino acid transporters ASCT2 and LAT1 in cancer: partners in crime?. Semin Cancer Biol.

[43] Nicklin P, Bergman P, Zhang B, Triantafellow E, Wang H, Nyfeler B (2009). Bidirectional transport of amino acids regulates mTOR and autophagy. Cell.

[44] Nachef M, Ali AK, Almutairi SM, Lee SH (2021). Targeting SLC1A5 and SLC3A2/SLC7A5 as a potential strategy to strengthen anti-tumor immunity in the tumor microenvironment. Front Immunol.

[45] Kurozumi S, Kaira K, Matsumoto H, Kurosumi M, Yokobori T, Kanai Y (2022). Association of L-type amino acid transporter 1 (LAT1) with the immune system and prognosis in invasive breast cancer. Sci Rep.

[46] Najumudeen AK, Ceteci F, Fey SK, Hamm G, Steven RT, Hall H (2021). The amino acid transporter SLC7A5 is required for efficient growth of KRAS-mutant colorectal cancer. Nat Genet.

[47] Wang J, Chen X, Su L, Li P, Liu B, Zhu Z (2013). LAT-1 functions as a promotor in gastric cancer associated with clinicopathologic features. Biomed Pharmacother.

[48] Liu YH, Li YL, Shen HT, Chien PJ, Sheu GT, Wang BY (2021). L-type amino acid transporter 1 regulates cancer stemness and the expression of programmed cell death 1 ligand 1 in lung cancer cells. Int J Mol Sci..

[49] Altan B, Kaira K, Watanabe A, Kubo N, Bao P, Dolgormaa G (2018). Relationship between LAT1 expression and resistance to chemotherapy in pancreatic ductal adenocarcinoma. Cancer Chemother Pharmacol.

[50] Xu M, Sakamoto S, Matsushima J, Kimura T, Ueda T, Mizokami A (2016). Up-regulation of LAT1 during antiandrogen therapy contributes to progression in prostate cancer cells. J Urol.

[51] Srisongkram T, Bahrami K, Jarvinen J, Timonen J, Rautio J, Weerapreeyakul N (2022). Development of sesamol carbamate-L-phenylalanine prodrug targeting L-Type Amino Acid Transporter1 (LAT1) as a potential antiproliferative agent against melanoma. Int J Mol Sci..

[52] Sato K, Miyamoto M, Takano M, Furuya K, Tsuda H (2019). Significant relationship between the LAT1 expression pattern and chemoresistance in ovarian clear cell carcinoma. Virchows Arch.

[53] Namikawa M, Kakizaki S, Kaira K, Tojima H, Yamazaki Y, Horiguchi N (2015). Expression of amino acid transporters (LAT1, ASCT2 and xCT) as clinical significance in hepatocellular carcinoma. Hepatol Res.

[54] Chen Z, Gao Y, Huang X, Yao Y, Chen K, Zeng S (2021). Tissue-based metabolomics reveals metabolic biomarkers and potential therapeutic targets for esophageal squamous cell carcinoma. J Pharm Biomed Anal.

[55] Sniegowski T, Korac K, Bhutia YD, Ganapathy V (2021). SLC6A14 and SLC38A5 drive the glutaminolysis and serine-glycine-one-carbon pathways in cancer. Pharmaceuticals (Basel)..

[56] Sloan JL, Mager S (1999). Cloning and functional expression of a human Na(+) and Cl(-)-dependent neutral and cationic amino acid transporter B(0+). J Biol Chem.

[57] Gupta N, Prasad PD, Ghamande S, Moore-Martin P, Herdman AV, Martindale RG (2006). Up-regulation of the amino acid transporter ATB(0,+) (SLC6A14) in carcinoma of the cervix. Gynecol Oncol.

[58] Karunakaran S, Ramachandran S, Coothankandaswamy V, Elangovan S, Babu E, Periyasamy-Thandavan S (2011). SLC6A14 (ATB0,+) protein, a highly concentrative and broad specific amino acid transporter, is a novel and effective drug target for treatment of estrogen receptor-positive breast cancer. J Biol Chem.

[59] Coothankandaswamy V, Cao S, Xu Y, Prasad PD, Singh PK, Reynolds CP (2016). Amino acid transporter SLC6A14 is a novel and effective drug target for pancreatic cancer. Br J Pharmacol.

[60] Guo Q, Xu W, Li X, Sun JL, Gu XC, Jing FB (2022). SLC6A14 depletion contributes to amino acid starvation to suppress EMT-induced metastasis in gastric cancer by perturbing the PI3K/AKT/mTORC1 pathway. Biomed Res Int.

[61] Taurino G, Chiu M, Bianchi MG, Griffini E, Bussolati O (2023). The SLC38A5/SNAT5 amino acid transporter: from pathophysiology to pro-cancer roles in the tumor microenvironment. Am J Physiol Cell Physiol.

[62] Wang K, Cao F, Fang W, Hu Y, Chen Y, Ding H (2013). Activation of SNAT1/SLC38A1 in human breast cancer: correlation with p-Akt overexpression. BMC Cancer.

[63] Xie J, Li P, Gao HF, Qian JX, Yuan LY, Wang JJ (2014). Overexpression of SLC38A1 is associated with poorer prognosis in Chinese patients with gastric cancer. BMC Gastroenterol.

[64] Zhou FF, Xie W, Chen SQ, Wang XK, Liu Q, Pan XK (2017). SLC38A1 promotes proliferation and migration of human colorectal cancer cells. J Huazhong Univ Sci Technolog Med Sci.

[65] Liu L, Su S, Ye D, Yu Z, Lu W, Li X (2022). Long non-coding RNA OGFRP1 regulates cell proliferation and ferroptosis by miR-299-3p/SLC38A1 axis in lung cancer. Anticancer Drugs.

[66] Liu Y, Yang Y, Jiang L, Xu H, Wei J (2021). High expression levels of SLC38A1 are correlated with poor prognosis and defective immune infiltration in hepatocellular carcinoma. J Oncol.

[67] Bohme-Schafer I, Lorentz S, Bosserhoff AK (2022). Role of amino acid transporter SNAT1/SLC38A1 in human melanoma. Cancers (Basel)..

[68] Okudaira H, Shikano N, Nishii R, Miyagi T, Yoshimoto M, Kobayashi M (2011). Putative transport mechanism and intracellular fate of trans-1-amino-3-18F-fluorocyclobutanecarboxylic acid in human prostate cancer. J Nucl Med.

[69] Sudo H, Tsuji AB, Sugyo A, Okada M, Kato K, Zhang MR (2018). Direct comparison of 2-amino[3-11C] isobutyric acid and 2-amino[11C]methyl-isobutyric acid uptake in eight lung cancer xenograft models. Int J Oncol.

[70] Zhao X, Jin L, Liu Y, Liu Z, Liu Q (2022). Bioinformatic analysis of the role of solute carrier-glutamine transporters in breast cancer. Ann Transl Med.

[71] Morotti M, Zois CE, El-Ansari R, Craze ML, Rakha EA, Fan SJ (2021). Increased expression of glutamine transporter SNAT2/SLC38A2 promotes glutamine dependence and oxidative stress resistance, and is associated with worse prognosis in triple-negative breast cancer. Br J Cancer.

[72] Morotti M, Bridges E, Valli A, Choudhry H, Sheldon H, Wigfield S (2019). Hypoxia-induced switch in SNAT2/SLC38A2 regulation generates endocrine resistance in breast cancer. Proc Natl Acad Sci U S A.

[73] Wang Y, Fu L, Cui M, Wang Y, Xu Y, Li M (2017). Amino acid transporter SLC38A3 promotes metastasis of non-small cell lung cancer cells by activating PDK1. Cancer Lett.

[74] Ramachandran S, S RS, Sharma M, Thangaraju M, V VS, Sneigowski T (2021). Expression and function of SLC38A5, an amino acid-coupled Na+/H+ exchanger, in triple-negative breast cancer and its relevance to macropinocytosis. Biochem J..

[75] Kim MJ, Kim HS, Kang HW, Lee DE, Hong WC, Kim JH (2023). SLC38A5 modulates ferroptosis to overcome gemcitabine resistance in pancreatic cancer. Cells-Basel..

[76] Shen X, Wang G, He H, Shang P, Yan B, Wang X (2024). SLC38A5 promotes glutamine metabolism and inhibits cisplatin chemosensitivity in breast cancer. Breast Cancer.

[77] Yang L, Venneti S, Nagrath D (2017). Glutaminolysis: a hallmark of cancer metabolism. Annu Rev Biomed Eng.

[78] Sellers K, Fox MP, Bousamra M, Slone SP, Higashi RM, Miller DM (2015). Pyruvate carboxylase is critical for non-small-cell lung cancer proliferation. J Clin Invest.

[79] Gaglio D, Soldati C, Vanoni M, Alberghina L, Chiaradonna F (2009). Glutamine deprivation induces abortive s-phase rescued by deoxyribonucleotides in k-ras transformed fibroblasts. PLoS One.

[80] Ward PS, Patel J, Wise DR, Abdel-Wahab O, Bennett BD, Coller HA (2010). The common feature of leukemia-associated IDH1 and IDH2 mutations is a neomorphic enzyme activity converting alpha-ketoglutarate to 2-hydroxyglutarate. Cancer Cell.

[81] Welbourne TC (1979). Ammonia production and glutamine incorporation into glutathione in the functioning rat kidney. Can J Biochem.

[82] Zack TI, Schumacher SE, Carter SL, Cherniack AD, Saksena G, Tabak B (2013). Pan-cancer patterns of somatic copy number alteration. Nat Genet.

[83] Yuneva M, Zamboni N, Oefner P, Sachidanandam R, Lazebnik Y (2007). Deficiency in glutamine but not glucose induces MYC-dependent apoptosis in human cells. J Cell Biol.

[84] Gao P, Tchernyshyov I, Chang TC, Lee YS, Kita K, Ochi T (2009). c-Myc suppression of miR-23a/b enhances mitochondrial glutaminase expression and glutamine metabolism. Nature.

[85] Wise DR, DeBerardinis RJ, Mancuso A, Sayed N, Zhang XY, Pfeiffer HK (2008). Myc regulates a transcriptional program that stimulates mitochondrial glutaminolysis and leads to glutamine addiction. Proc Natl Acad Sci U S A.

[86] Anso E, Mullen AR, Felsher DW, Mates JM, Deberardinis RJ, Chandel NS (2013). Metabolic changes in cancer cells upon suppression of MYC. Cancer Metab.

[87] Jeong SM, Lee A, Lee J, Haigis MC (2014). SIRT4 protein suppresses tumor formation in genetic models of Myc-induced B cell lymphoma. J Biol Chem.

[88] Shroff EH, Eberlin LS, Dang VM, Gouw AM, Gabay M, Adam SJ (2015). MYC oncogene overexpression drives renal cell carcinoma in a mouse model through glutamine metabolism. Proc Natl Acad Sci U S A.

[89] Dejure FR, Royla N, Herold S, Kalb J, Walz S, Ade CP (2017). The MYC mRNA 3'-UTR couples RNA polymerase II function to glutamine and ribonucleotide levels. EMBO J.

[90] Munksgaard Thoren M, Vaapil M, Staaf J, Planck M, Johansson ME, Mohlin S (2017). Myc-induced glutaminolysis bypasses HIF-driven glycolysis in hypoxic small cell lung carcinoma cells. Oncotarget.

[91] Kandasamy P, Zlobec I, Nydegger DT, Pujol-Gimenez J, Bhardwaj R, Shirasawa S (2021). Oncogenic KRAS mutations enhance amino acid uptake by colorectal cancer cells via the hippo signaling effector YAP1. Mol Oncol.

[92] Zhu Q, Zhou H, Wu L, Lai Z, Geng D, Yang W (2022). O-GlcNAcylation promotes pancreatic tumor growth by regulating malate dehydrogenase 1. Nat Chem Biol.

[93] Meijer TWH, Looijen-Salamon MG, Lok J, van den Heuvel M, Tops B, Kaanders J (2019). Glucose and glutamine metabolism in relation to mutational status in NSCLC histological subtypes. Thorac Cancer.

[94] Saxton RA, Sabatini DM (2017). mTOR signaling in growth, metabolism, and disease. Cell.

[95] Grzmil M, Wiesmann F, Schibli R, Behe M (2022). Targeting mTORC1 activity to improve efficacy of radioligand therapy in cancer. Cancers (Basel)..

[96] Ni R, Li Z, Li L, Peng D, Ming Y, Li L (2023). Rethinking glutamine metabolism and the regulation of glutamine addiction by oncogenes in cancer. Front Oncol.

[97] Choo AY, Kim SG, Vander Heiden MG, Mahoney SJ, Vu H, Yoon SO (2010). Glucose addiction of TSC null cells is caused by failed mTORC1-dependent balancing of metabolic demand with supply. Mol Cell.

[98] Csibi A, Fendt SM, Li C, Poulogiannis G, Choo AY, Chapski DJ (2013). The mTORC1 pathway stimulates glutamine metabolism and cell proliferation by repressing SIRT4. Cell.

[99] Csibi A, Lee G, Yoon SO, Tong H, Ilter D, Elia I (2014). The mTORC1/S6K1 pathway regulates glutamine metabolism through the eIF4B-dependent control of c-Myc translation. Curr Biol.

[100] Chen M, Wang G, Xu Z, Sun J, Liu B, Chang L, et al. Loss of RACK1 promotes glutamine addiction via activating AKT/mTOR/ASCT2 axis to facilitate tumor growth in gastric cancer. Cell Oncol (Dordr). 2024;47(1):113–28. 10.1007/s13402-023-00854-1 PMC12974089 37578594

[101] Hao Y, Samuels Y, Li Q, Krokowski D, Guan BJ, Wang C (2016). Oncogenic PIK3CA mutations reprogram glutamine metabolism in colorectal cancer. Nat Commun.

[102] Oh DY, Bang YJ (2020). HER2-targeted therapies - a role beyond breast cancer. Nat Rev Clin Oncol.

[103] Qie S, Chu C, Li W, Wang C, Sang N (2014). ErbB2 activation upregulates glutaminase 1 expression which promotes breast cancer cell proliferation. J Cell Biochem.

[104] Hu X, Ma Z, Xu B, Li S, Yao Z, Liang B (2023). Glutamine metabolic microenvironment drives M2 macrophage polarization to mediate trastuzumab resistance in HER2-positive gastric cancer. Cancer Commun (Lond).

[105] Xiao-Yan W, Xiao-Xia Y, Peng-Fei S, Zong-Xue Z, Xiu-Li G (2023). Metabolic reprogramming of glutamine involved in tumorigenesis, multidrug resistance and tumor immunity. Eur J Pharmacol.

[106] Suzuki S, Tanaka T, Poyurovsky MV, Nagano H, Mayama T, Ohkubo S (2010). Phosphate-activated glutaminase (GLS2), a p53-inducible regulator of glutamine metabolism and reactive oxygen species. Proc Natl Acad Sci U S A.

[107] Zhan H, Ciano K, Dong K, Zucker S (2015). Targeting glutamine metabolism in myeloproliferative neoplasms. Blood Cells Mol Dis.

[108] Lukey MJ, Greene KS, Erickson JW, Wilson KF, Cerione RA (2016). The oncogenic transcription factor c-Jun regulates glutaminase expression and sensitizes cells to glutaminase-targeted therapy. Nat Commun.

[109] Baenke F, Chaneton B, Smith M, Van Den Broek N, Hogan K, Tang H (2016). Resistance to BRAF inhibitors induces glutamine dependency in melanoma cells. Mol Oncol.

[110] Garcia-Cao I, Song MS, Hobbs RM, Laurent G, Giorgi C, de Boer VC (2012). Systemic elevation of PTEN induces a tumor-suppressive metabolic state. Cell.

[111] Reynolds MR, Lane AN, Robertson B, Kemp S, Liu Y, Hill BG (2014). Control of glutamine metabolism by the tumor suppressor Rb. Oncogene.

[112] Lee SY, Jeon HM, Ju MK, Jeong EK, Kim CH, Park HG (2016). Dlx-2 and glutaminase upregulate epithelial-mesenchymal transition and glycolytic switch. Oncotarget.

[113] Newsholme P. Why is L-glutamine metabolism important to cells of the immune system in health, postinjury, surgery or infection? J Nutr. 2001;131(9):2515S–22S. 10.1093/jn/131.9.2515S 11533304

[114] Cruzat V, MacedoRogero M, Noel Keane K, Curi R, Newsholme P (2018). Glutamine: metabolism and immune function, supplementation and clinical translation. Nutrients..

[115] Mills EL, Kelly B, O’Neill LAJ (2017). Mitochondria are the powerhouses of immunity. Nat Immunol.

[116] Ardawi MS, Newsholme EA (1984). Intracellular localization and properties of phosphate-dependent glutaminase in rat mesenteric lymph nodes. Biochem J.

[117] Jensen H, Potempa M, Gotthardt D, Lanier LL (2017). Cutting edge: IL-2-induced expression of the amino acid transporters SLC1A5 and CD98 is a prerequisite for NKG2D-mediated activation of human NK cells. J Immunol.

[118] Maciolek JA, Pasternak JA, Wilson HL (2014). Metabolism of activated T lymphocytes. Curr Opin Immunol.

[119] Nakaya M, Xiao Y, Zhou X, Chang JH, Chang M, Cheng X (2014). Inflammatory T cell responses rely on amino acid transporter ASCT2 facilitation of glutamine uptake and mTORC1 kinase activation. Immunity.

[120] Huang H, Zhou P, Wei J, Long L, Shi H, Dhungana Y (2021). In vivo CRISPR screening reveals nutrient signaling processes underpinning CD8(+) T cell fate decisions. Cell..

[121] Jiang S, Yan W, Wang SE, Baltimore D (2018). Let-7 suppresses B cell activation through restricting the availability of necessary nutrients. Cell Metab..

[122] Biswas SK, Mantovani A (2010). Macrophage plasticity and interaction with lymphocyte subsets: cancer as a paradigm. Nat Immunol..

[123] Liu PS, Wang H, Li X, Chao T, Teav T, Christen S, et al. α-ketoglutarate orchestrates macrophage activation through metabolic and epigenetic reprogramming. Nat Immunol. 2017. 10.1038/ni.3796 28714978

[124] Newsholme Philip. Why is l-glutamine metabolism important to cells of the immune system in health, postinjury, surgery or infection? J Nutr. 2001. 10.1093/jn/131.9.2515S 11533304

[125] Ren W, Xia Y, Chen S, Wu G, Bazer FW, Zhou B (2019). Glutamine metabolism in macrophages: a novel target for obesity/type 2 diabetes. Adv Nutr.

[126] Liu PS, Chen YT, Li X, Hsueh PC, Tzeng SF, Chen H (2023). CD40 signal rewires fatty acid and glutamine metabolism for stimulating macrophage anti-tumorigenic functions. Nat Immunol.

[127] Loftus RM, Assmann N, Kedia-Mehta N, O'Brien KL, Garcia A, Gillespie C (2018). Amino acid-dependent cMyc expression is essential for NK cell metabolic and functional responses in mice. Nat Commun.

[128] Presnell SR, Spear HK, Durham J, Riddle T, Applegate A, Lutz CT (2020). Differential fuel requirements of human NK cells and human CD8 T cells: glutamine regulates glucose uptake in strongly activated CD8 T cells. Immunohorizons.

[129] Ricciardi S, Manfrini N, Alfieri R, Calamita P, Crosti MC, Gallo S (2018). The translational machinery of human CD4(+) T cells is poised for activation and controls the switch from quiescence to metabolic remodeling. Cell Metab..

[130] Sinclair LV, Rolf J, Emslie E, Shi Y-B, Taylor PM, Cantrell DA (2013). Control of amino-acid transport by antigen receptors coordinates the metabolic reprogramming essential for T cell differentiation. Nat Immunol.

[131] Carr EL, Kelman A, Wu GS, Gopaul R, Senkevitch E, Aghvanyan A (2010). Glutamine uptake and metabolism are coordinately regulated by ERK/MAPK during T lymphocyte activation. J Immunol.

[132] Lee K, Thompson EA, Gharaie S, Patel CH, Kurzhagen JT, Pierorazio PM (2023). T cell metabolic reprogramming in acute kidney injury and protection by glutamine blockade. JCI Insight..

[133] Madi A, Weisshaar N, Buettner M, Poschet G, Ma S, Wu J (2022). CD8 agonism functionally activates memory T cells and enhances antitumor immunity. Int J Cancer.

[134] Edwards DN, Ngwa VM, Raybuck AL, Wang S, Hwang Y, Kim LC (2021). Selective glutamine metabolism inhibition in tumor cells improves antitumor T lymphocyte activity in triple-negative breast cancer. J Clin Invest..

[135] Sakai C, Nishikawa H (2018). Immunosuppressive environment in tumors. Gan To Kagaku Ryoho.

[136] Yang G, Xia Y, Ren W (2021). Glutamine metabolism in Th17/Treg cell fate: applications in Th17 cell-associated diseases. Sci China Life Sci.

[137] Kumar A, Yarosz EL, Andren A, Zhang L, Lyssiotis CA, Chang CH (2022). NKT cells adopt a glutamine-addicted phenotype to regulate their homeostasis and function. Cell Rep.

[138] Crawford J, Cohen HJ (1985). The essential role of L-glutamine in lymphocyte differentiation in vitro. J Cell Physiol.

[139] Le A, Lane AN, Hamaker M, Bose S, Gouw A, Barbi J (2012). Glucose-independent glutamine metabolism via TCA cycling for proliferation and survival in B cells. Cell Metab.

[140] Waters LR, Ahsan FM, Wolf DM, Shirihai O, Teitell MA (2018). Initial B cell activation induces metabolic reprogramming and mitochondrial remodeling. iScience..

[141] Zhang X, Wang G, Bi Y, Jiang Z, Wang X (2022). Inhibition of glutaminolysis ameliorates lupus by regulating T and B cell subsets and downregulating the mTOR/P70S6K/4EBP1 and NLRP3/caspase-1/IL-1beta pathways in MRL/lpr mice. Int Immunopharmacol.

[142] Mielle J, Morel J, Elhmioui J, Combe B, Macia L, Dardalhon V (2022). Glutamine promotes the generation of B10(+) cells via the mTOR/GSK3 pathway. Eur J Immunol.

[143] Mantovani A, Cassatella MA, Costantini C, Jaillon S (2011). Neutrophils in the activation and regulation of innate and adaptive immunity. Nat Rev Immunol.

[144] Pithon-Curi TC, De Melo MP, Curi R (2004). Glucose and glutamine utilization by rat lymphocytes, monocytes and neutrophils in culture: a comparative study. Cell Biochem Funct.

[145] Pithon-Curi TC, Trezena AG, Tavares-Lima W, Curi R (2002). Evidence that glutamine is involved in neutrophil function. Cell Biochem Funct.

[146] Lagranha CJ, Senna SM, de Lima TM, Silva E, Doi SQ, Curi R (2004). Beneficial effect of glutamine on exercise-induced apoptosis of rat neutrophils. Med Sci Sports Exerc..

[147] Oh MH, Sun IH, Zhao L, Leone RD, Sun IM, Xu W (2020). Targeting glutamine metabolism enhances tumor-specific immunity by modulating suppressive myeloid cells. J Clin Investig.

[148] Kao KC, Vilbois S, Tsai CH, Ho PC (2022). Metabolic communication in the tumour-immune microenvironment. Nat Cell Biol.

[149] Wilmore DW, Shabert JK (1998). Role of glutamine in immunologic responses. Nutrition..

[150] Singer K, Cheng WC, Kreutz M, Ho PC, Siska PJ (2018). Immunometabolism in cancer at a glance. Dis Model Mech..

[151] Chen JF, Wang R, Liu ZL, Fan J, Liu SL, Tan SD (2022). Unbalanced glutamine partitioning between CD8T cells and cancer cells accompanied by immune cell dysfunction in hepatocellular carcinoma. Cells-Basel..

[152] Chang CH, Qiu J, O'Sullivan D, Buck MD, Noguchi T, Curtis JD (2015). Metabolic competition in the tumor microenvironment is a driver of cancer progression. Cell.

[153] Chen P, Han Y, Wang L, Zheng Y, Zhu Z, Zhao Y (2023). Spatially resolved metabolomics combined with the 3d tumor-immune cell coculture spheroid highlights metabolic alterations during antitumor immune response. Anal Chem.

[154] Zhang X, Halberstam AA, Zhu W, Leitner BP, Thakral D, Bosenberg MW (2022). Isotope tracing reveals distinct substrate preference in murine melanoma subtypes with differing anti-tumor immunity. Cancer Metab.

[155] Kondo M, Kumagai S, Nishikawa H. Metabolic advantages of regulatory T cells dictated by cancer cells. Int Immunol. 2024;36(2):75–86. 10.1093/intimm/dxad035 37837615

[156] Tsai CC, Tiao MM, Sheen JM, Huang LT, Tain YL, Lin IC (2019). Obesity programmed by prenatal dexamethasone and postnatal high-fat diet leads to distinct alterations in nutrition sensory signals and circadian-clock genes in visceral adipose tissue. Lipids Health Dis.

[157] Battu S, Minhas G, Mishra A, Khan N (2017). Amino acid sensing via general control nonderepressible-2 kinase and immunological programming. Front Immunol.

[158] Willinger T, Staron M, Ferguson SM, De Camilli P, Flavell RA (2015). Dynamin 2-dependent endocytosis sustains T-cell receptor signaling and drives metabolic reprogramming in T lymphocytes. Proc Natl Acad Sci U S A.

[159] Shi H, Chapman NM, Wen J, Guy C, Long L, Dhungana Y (2019). Amino acids license kinase mTORC1 activity and treg cell function via small G proteins rag and rheb. Immunity..

[160] Metzler B, Gfeller P, Guinet E (2016). Restricting glutamine or glutamine-dependent purine and pyrimidine syntheses promotes human T cells with high FOXP3 expression and regulatory properties. J Immunol.

[161] Liu JQ, Geng XR, Hu TY, Mo LH, Luo XQ, Qiu SY (2022). Glutaminolysis is required in maintaining immune regulatory functions in B cells. Mucosal Immunol.

[162] Loftus RM, Assmann N, Kedia-Mehta N, O'Brien KL, Garcia A, Gillespie C (2018). Amino acid-dependent cMyc expression is essential for NK cell metabolic and functional responses in mice. Nat Commun..

[163] Nishino M, Ramaiya NH, Hatabu H, Hodi FS (2017). Monitoring immune-checkpoint blockade: response evaluation and biomarker development. Nat Rev Clin Oncol.

[164] Goodman A, Patel SP, Kurzrock R (2017). PD-1-PD-L1 immune-checkpoint blockade in B-cell lymphomas. Nat Rev Clin Oncol.

[165] Zheng Y, Yao Y, Ge T, Ge S, Jia R, Song X (2023). Amino acid metabolism reprogramming: shedding new light on T cell anti-tumor immunity. J Exp Clin Cancer Res.

[166] Byun JK, Park M, Lee S, Yun JW, Lee J, Kim JS (2020). Inhibition of glutamine utilization synergizes with immune checkpoint inhibitor to promote antitumor immunity. Mol Cell..

[167] Xu Y, He L, Fu Q, Hu J (2021). Metabolic reprogramming in the tumor microenvironment with immunocytes and immune checkpoints. Front Oncol.

[168] Ma G, Liang Y, Chen Y, Wang L, Li D, Liang Z (2020). Glutamine deprivation induces PD-L1 expression via activation of EGFR/ERK/c-jun signaling in renal cancer. Mol Cancer Res.

[169] Wang L, Xu T, Yang X, Liang Z, Zhang J, Li D (2021). Immunosuppression induced by glutamine deprivation occurs via activating PD-L1 transcription in bladder cancer. Front Mol Biosci.

[170] Byun JK, Park M, Lee S, Yun JW, Lee J, Kim JS (2020). Inhibition of glutamine utilization synergizes with immune checkpoint inhibitor to promote antitumor immunity. Mol Cell..

[171] Zhao L, Rao X, Zheng R, Huang C, Kong R, Yu X (2023). Targeting glutamine metabolism with photodynamic immunotherapy for metastatic tumor eradication. J Control Release.

[172] Gao Y, Zhang H, Tang L, Li F, Yang L, Xiao H, et al. Cancer Nanobombs Delivering Artoxplatin with a Polyigniter Bearing Hydrophobic Ferrocene Units Upregulate PD-L1 Expression and Stimulate Stronger Anticancer Immunity. Adv Sci (Weinh). 2024;11(4):e2300806. 10.1002/advs.202300806 PMC10811492 37166035

[173] Namkoong J, Shin SS, Lee HJ, Marín YE, Wall BA, Goydos JS (2007). Metabotropic glutamate receptor 1 and glutamate signaling in human melanoma. Can Res.

[174] Best SA, Gubser PM, Sethumadhavan S, Kersbergen A, Abril YLN, Goldford J (2022). Glutaminase inhibition impairs CD8 T cell activation in STK11-/Lkb1-deficient lung cancer. Cell Metab..

[175] Xiong TT, He P, Zhou M, Zhong D, Yang T, He WH (2022). Glutamate blunts cell-killing effects of neutrophils in tumor microenvironment. Cancer Sci.

[176] Robert SM, Sontheimer H (2014). Glutamate transporters in the biology of malignant gliomas. Cell Mol Life Sci.

[177] Morikawa N, Tachibana M, Ago Y, Goda H, Sakurai F, Mizuguchi H (2018). LY341495, an mGluR2/3 antagonist, regulates the immunosuppressive function of myeloid-derived suppressor cells and inhibits melanoma tumor growth. Biol Pharm Bull.

[178] Pacheco R, Ciruela F, Casadó V, Mallol J, Gallart T, Lluis C (2004). Group I metabotropic glutamate receptors mediate a dual role of glutamate in T cell activation. J Biol Chem.

[179] Siska PJ, Kim B, Ji XM, Hoeksema MD, Massion PP, Beckermann KE (2016). Fluorescence-based measurement of cystine uptake through xCT shows requirement for ROS detoxification in activated lymphocytes. J Immunol Methods.

[180] Koda S, Hu J, Ju XM, Sun GW, Shao SM, Tang RX (2023). The role of glutamate receptors in the regulation of the tumor microenvironment. Front Immunol..

[181] Lukey MJ, Wilson KF, Cerione RA (2013). Therapeutic strategies impacting cancer cell glutamine metabolism. Future Med Chem.

[182] Magill GB, Myers WP, Reilly HC, Putnam RC, Magill JW, Sykes MP (1957). Pharmacological and initial therapeutic observations on 6-diazo-5-oxo-1-norleucine (DON) in human neoplastic disease. Cancer.

[183] Sharma NS, Gupta VK, Garrido VT, Hadad R, Durden BC, Kesh K (2020). Targeting tumor-intrinsic hexosamine biosynthesis sensitizes pancreatic cancer to anti-PD1 therapy. J Clin Invest.

[184] Frejlachova A, Lencova R, Venhauerova A, Skalickova M, Uher O, Caisova V (2023). The combination of immunotherapy and a glutamine metabolism inhibitor represents an effective therapeutic strategy for advanced and metastatic murine pancreatic adenocarcinoma. Int Immunopharmacol.

[185] Huang M, Xiong D, Pan J, Zhang Q, Sei S, Shoemaker RH (2022). Targeting glutamine metabolism to enhance immunoprevention of EGFR-driven lung cancer. Adv Sci (Weinh).

[186] Yokoyama Y, Estok TM, Wild R (2022). Sirpiglenastat (DRP-104) induces antitumor efficacy through direct, broad antagonism of glutamine metabolism and stimulation of the innate and adaptive immune systems. Mol Cancer Ther.

[187] Rais R, Lemberg KM, Tenora L, Arwood ML, Pal A, Alt J (2022). Discovery of DRP-104, a tumor-targeted metabolic inhibitor prodrug. Sci Adv..

[188] Pillai R, LeBoeuf SE, Hao Y, New C, Blum JLE, Rashidfarrokhi A, et al. Glutamine antagonist DRP-104 suppresses tumor growth and enhances response to checkpoint blockade in KEAP1 mutant lung cancer. bioRxiv. 2023. 10.1126/sciadv.adm9859 PMC10971495 38536921

[189] Jin L, Alesi GN, Kang S (2016). Glutaminolysis as a target for cancer therapy. Oncogene.

[190] Kanai Y, Hediger MA (2004). The glutamate/neutral amino acid transporter family SLC1: molecular, physiological and pharmacological aspects. Pflugers Arch.

[191] Schulte ML, Fu A, Zhao P, Li J, Geng L, Smith ST (2018). Pharmacological blockade of ASCT2-dependent glutamine transport leads to antitumor efficacy in preclinical models. Nat Med.

[192] Han L, Zhou J, Li L, Wu X, Shi Y, Cui W (2022). SLC1A5 enhances malignant phenotypes through modulating ferroptosis status and immune microenvironment in glioma. Cell Death Dis.

[193] Li Q, Zhong X, Yao W, Yu J, Wang C, Li Z (2022). Inhibitor of glutamine metabolism V9302 promotes ROS-induced autophagic degradation of B7H3 to enhance antitumor immunity. J Biol Chem.

[194] Tang Y, Wang S, Li Y, Yuan C, Zhang J, Xu Z (2022). Simultaneous glutamine metabolism and PD-L1 inhibition to enhance suppression of triple-negative breast cancer. J Nanobiotechnol.

[195] Scalise M, Pochini L, Galluccio M, Console L, Indiveri C (2017). Glutamine transport and mitochondrial metabolism in cancer cell growth. Front Oncol.

[196] Huang R, Wang H, Hong J, Wu J, Huang O, He J (2023). Targeting glutamine metabolic reprogramming of SLC7A5 enhances the efficacy of anti-PD-1 in triple-negative breast cancer. Front Immunol.

[197] Chapman VM, Ruddle FH (1972). Glutamate oxaloacetate transaminase (got) genetics in the mouse: polymorphism of got-1. Genetics.

[198] Meléndez-Rodríguez F, Urrutia AA, Lorendeau D, Rinaldi G, Roche O, Böğürcü-Seidel N (2019). HIF1a suppresses tumor cell proliferation through inhibition of aspartate biosynthesis. Cell Rep.

[199] Varghese S, Pramanik S, Williams LJ, Hodges HR, Hudgens CW, Fischer GM (2021). The glutaminase inhibitor CB-839 (Telaglenastat) enhances the antimelanoma activity of t-cell-mediated immunotherapies. Mol Cancer Ther.

[200] Best SA, Gubser PM, Sethumadhavan S, Kersbergen A, Negron Abril YL, Goldford J (2022). Glutaminase inhibition impairs CD8 T cell activation in STK11-/Lkb1-deficient lung cancer. Cell Metab..

[201] Zhang R, Li R, Zhang L, Chen G, Mo L, Jiang R (2023). A dual-mechanism based nutrient partitioning nanoregulator for enhanced immunotherapy against anti-PD-1 resistant tumors. ACS Nano.

[202] Wang JJ, Siu MK, Jiang YX, Leung TH, Chan DW, Wang HG (2021). A combination of glutaminase inhibitor 968 and PD-L1 blockade boosts the immune response against ovarian cancer. Biomolecules..

[203] Mai Z, Zhong J, Zhang J, Chen G, Tang Y, Ma W, et al. Carrier-free immunotherapeutic nano-booster with dual synergistic effects based on glutaminase inhibition combined with photodynamic therapy. ACS Nano. 2023;17(2):1583–96. 10.1021/acsnano.2c11037 36595443

[204] Jin XK, Zhang SM, Liang JL, Zhang SK, Qin YT, Huang QX, et al. A PD-L1-targeting regulator for metabolic reprogramming to enhance glutamine inhibition-mediated synergistic antitumor metabolic and immune therapy. Adv Mater. 2023:36(6):e2309094. 10.1002/adma.202309094 38014890

[205] Zhang J, Wei L, Ma X, Wang J, Liang S, Chen K (2023). pH-sensitive tumor-tropism hybrid membrane-coated nanoparticles for reprogramming the tumor microenvironment and boosting the antitumor immunity. Acta Biomater.

[206] Ren J, Zhou J, Liu H, Jiao X, Cao Y, Xu Z (2021). Ultrasound (US)-activated redox dyshomeostasis therapy reinforced by immunogenic cell death (ICD) through a mitochondrial targeting liposomal nanosystem. Theranostics.

[207] Kang J, Chun J, Hwang JS, Pan C, Li J, Boese AC (2022). EGFR-phosphorylated GDH1 harmonizes with RSK2 to drive CREB activation and tumor metastasis in EGFR-activated lung cancer. Cell Rep.

[208] Abrego J, Sanford-Crane H, Oon C, Xiao X, Betts CB, Sun D (2022). A cancer cell-intrinsic GOT2-PPARdelta axis suppresses antitumor immunity. Cancer Discov.

[209] Wang B, Pei J, Xu S, Liu J, Yu J (2023). System analysis based on glutamine catabolic-related enzymes identifies GPT2 as a novel immunotherapy target for lung adenocarcinoma. Comput Biol Med.

[210] Kim M, Gwak J, Hwang S, Yang S, Jeong SM (2019). Mitochondrial GPT2 plays a pivotal role in metabolic adaptation to the perturbation of mitochondrial glutamine metabolism. Oncogene.

[211] Yang Y, He P, Hou Y, Liu Z, Zhang X, Li N (2022). Osmundacetone modulates mitochondrial metabolism in non-small cell lung cancer cells by hijacking the glutamine/glutamate/alpha-KG metabolic axis. Phytomedicine.

[212] Wang ES, Frankfurt O, Orford KW, Bennett M, Konopleva M (2015). Phase 1 study of CB-839, a first-in-class, orally administered small molecule inhibitor of glutaminase in patients with relapsed/refractory leukemia. Blood.

[213] Vogl DT, Younes A, Stewart K, Orford KW, Berdeja JG (2015). Phase 1 study of CB-839, a first-in-class, glutaminase inhibitor in patients with multiple myeloma and lymphoma. Blood.

[214] Harding JJ, Telli ML, Munster PN, Le MH, Molineaux C, Bennett MK, et al. Safety and tolerability of increasing doses of CB-839, a first-in-class, orally administered small molecule inhibitor of glutaminase, in solid tumors. J Clin Oncol. 2015;33(15):2512.

[215] DeMichele A, Harding JJ, Telli ML, Munster PN, Mckay R, Iliopoulos O, et al. Phase 1 study of CB-839, a small molecule inhibitor of glutaminase (GLS) in combination with paclitaxel (Pac) in patients (pts) with triple negative breast cancer (TNBC). J Clin Oncol. 2016;34(15):1011.

[216] Meric-Bernstam F, Lee RJ, Carthon BC, Iliopoulos O, Mier JW, Patel MR, et al. CB-839, a glutaminase inhibitor, in combination with cabozantinib in patients with clear cell and papillary metastatic renal cell cancer (mRCC): results of a phase i study. J Clin Oncol. 2019;37(7):549.

[217] Meric-Bernstam F, Tannir N, Harding J, Voss M, Mier J, DeMichele A (2016). 26 - Phase 1 study of CB-839, a small molecule inhibitor of glutaminase, in combination with everolimus in patients (pts) with clear cell and papillary renal cell cancer (RCC). Eur J Cancer.

[218] Tannir NM, Agarwal N, Dawson NA, Motzer RJ, Jacobs CM, Choueiri TK, et al. CANTATA: Randomized, international, double-blind study of CB-839 plus cabozantinib versus cabozantinib plus placebo in patients with metastatic renal cell carcinoma. J Clin Oncol. 2019;37(7):TPS682.

[219] Wang D, Li X, Gong G, Lu Y, Guo Z, Chen R (2023). An updated patent review of glutaminase inhibitors (2019–2022). Expert Opin Ther Pat.

[220] Soth MJ, Le K, Di Francesco ME, Hamilton MM, Liu G, Burke JP (2020). Discovery of IPN60090, a clinical stage selective glutaminase-1 (GLS-1) inhibitor with excellent pharmacokinetic and physicochemical properties. J Med Chem.

[221] Fu Y, Liu S, Zeng S, Shen H (2019). From bench to bed: the tumor immune microenvironment and current immunotherapeutic strategies for hepatocellular carcinoma. J Exp Clin Cancer Res.

[222] Jing X, Yang F, Shao C, Wei K, Xie M, Shen H (2019). Role of hypoxia in cancer therapy by regulating the tumor microenvironment. Mol Cancer.

[223] Leone RD, Powell JD (2020). Metabolism of immune cells in cancer. Nat Rev Cancer.

[224] Madden MZ, Ye X, Chi C, Fisher EL, Wolf MM, Needle GA (2023). Differential effects of glutamine inhibition strategies on antitumor CD8 T cells. J Immunol.

[225] Ahluwalia GS, Grem JL, Hao Z, Cooney DA (1990). Metabolism and action of amino acid analog anti-cancer agents. Pharmacol Ther.

