# Correction: A glutamine tug-of-war between cancer and immune cells: recent advances in unraveling the ongoing battle

**DOI:** 10.1186/s13046-024-03018-7

**Published:** 2024-03-26

**Authors:** Bolin Wang, Jinli Pei, Shengnan Xu, Jie Liu, Jinming Yu

**Affiliations:** 1 Lung Cancer Center, West China Hospital, Sichuan University, Chengdu, 610041 China; 2 Department of Radiation Oncology and Shandong ProvincialKey Laboratory of Radiation Oncology, Shandong Cancer Hospital and Institute, Shandong First Medical University and Shandong Academyof Medical Sciences, Jinan, Shandong, China; 3 Research Unit of Radiation Oncology, Chinese Academy of Medical Sciences, Jinan, Shandong, China

**Correction: J Exp Clin Cancer Res (2024) 43:74**

10.1186/s13046-024-02994-0

Following publication of the original article [1], errors were spotted particularly in Funding section. The correct funding statement is stated below:

**Incorrect Funding**

This work was supported by the National Natural Science Foundation of China (Grant Nos. 81,627,901, 82,203,014, 81,972,863, (Grant Nos. 81,627,901, 82,203,014, 81,972,863, 82,030,082, and 22,307,068), the Radiation Oncology Innovate Unit, Chinese Academy of Medical Sciences (Grant No. 2019RU071), the Natural Science Foundation of Shandong Province (Grant Nos. ZR2022QH017 and ZR2022QB015), the Academic Promotion Program of Shandong First Medical University (Grant No. 2019ZL002), the China Postdoctoral Science Foundation (Grant No. 2023M742149), and the Taishan Scholars Program (Grant No. tsqn202306386).

**Correct Funding**

This work was supported by the National Natural Science Foundation of China (Grant Nos. 81,627,901), the National Natural Science Foundation of China (Grant Nos. 82,203,014, 81,972,863, 82,030,082, and 22,307,068), the Radiation Oncology Innovate Unit, Chinese Academy of Medical Sciences (Grant No. 2019RU071), the Natural Science Foundation of Shandong Province (Grant Nos. ZR2022QH017 and ZR2022QB015), the Academic Promotion Program of Shandong First Medical University (Grant No. 2019ZL002), the China Postdoctoral Science Foundation (Grant No. 2023M742149), and the Taishan Scholars Program (Grant No. tsqn202306386).

The corrections do not affect the overall result or conclusion of the article. The original article has been corrected.
